# Evaluation of the MyFertiCoach Lifestyle App for Subfertile Couples: Single-Center Evaluation of Augmented Standard Care

**DOI:** 10.2196/64239

**Published:** 2025-03-10

**Authors:** Jesper Smeenk, Ellen Smit, Marc Jacobs, Ilse van Rooij

**Affiliations:** 1 Elisabeth TweeSteden Ziekenhuis Tilburg The Netherlands; 2 Ferring BV Hoofddorp The Netherlands; 3 MSJ Advies Helmond The Netherlands

**Keywords:** fertility, mHealth, pregnancy, lifestyle, app, smartphone

## Abstract

**Background:**

Many couples undergoing fertility treatment face multiple lifestyle risk factors that lower their chances of achieving pregnancy. The MyFertiCoach (MFC) app was designed as an integrated lifestyle program featuring modules on healthy weight management, nutrition, exercise, quitting smoking, reducing alcohol and drug use, and managing stress. We hypothesized that supplementing standard care with the MFC app would improve lifestyle outcomes.

**Objective:**

This study aims to assess the impact of the MFC app on changing multiple lifestyle habits in women seeking fertility treatment. The primary outcome is the change in the total risk score (TRS) at 3- and six-month follow-ups. The TRS is calculated for each individual as the sum of all risk scores per behavior (eg, vegetable/fruit/folic acid intake, smoking, and alcohol use) at 3 and 6 months. A higher TRS indicates unhealthier nutrition and lifestyle habits and a lower likelihood of achieving pregnancy. The secondary endpoints include changes in BMI, activity score, preconception dietary risk score, distress score (eg, perceived burden), smoking habits, alcohol intake, and program adherence.

**Methods:**

This retrospective, observational, single-center evaluation included patients between January 1, 2022, and December 31, 2023. Subfertile female patients aged 18-43 years and their partners, who were referred to a gynecologist, were invited to participate in online lifestyle coaching via the MFC app. The gynecologist selected relevant lifestyle modules based on the results of integrated screening questionnaires. We used (hierarchical) linear mixed models (LMMs) to estimate changes in outcomes. For missing data patterns deemed missing not at random, joint modeling was applied. Statistical significance was set at *P*≤.05, with methods in place to maintain the same false-positive rate.

**Results:**

A total of 1805 patients were invited to participate in the evaluation, with an average of 737 (40.83%) completing the screening questionnaire at baseline. For the TRS, 798 (44.21%) patients were included at baseline, of whom 517 (64.8%) involved their partner. On average, 282 of 744 (37.9%) patients submitted at least one follow-up questionnaire. Patients rated the app above average (n=137, median score of 7 on a 1-10 scale) on days 7 and 14. The TRS decreased by an average of 1.5 points (*P*<.001) at T3 and T6 compared with baseline, a clinically meaningful improvement. All secondary outcomes showed statistically significant positive changes for patients who used a relevant lifestyle module (*P*<.001). Most improvements were achieved by 3 months and remained significant at 6 months (*P*<.001), except for alcohol intake (*P*<.53). These findings were consistent across both LMMs and joint models.

**Conclusions:**

Our evaluation of a mobile health app integrated into standard care demonstrates immediate and clinically meaningful improvements in key lifestyle parameters among women seeking to become pregnant. Additional scientific research is needed to identify the causal pathways leading to sustained effectiveness. To maintain and enhance these outcomes, further tailoring of patient-specific programs is essential.

## Introduction

Many couples undergoing fertility treatment have multiple lifestyle risk factors, such as obesity, unbalanced nutritional habits, low physical activity, and alcohol consumption or smoking. These habits not only increase the risk of cardiovascular and metabolic diseases but also reduce the chance of becoming pregnant [1-4]. Reducing any or all of these lifestyle factors before pregnancy or assisted reproductive technology treatment can improve reproductive health [5-9]. In summary, the more negative lifestyle factors present, the lower the chance of becoming pregnant and the longer the time until pregnancy [10].

For instance, improving dietary patterns during the preconception period reduces the risk of several adverse birth outcomes, such as fetal growth restriction and babies born small or large for gestational age. Maternal complications, including gestational diabetes, hypertensive disorders, and premature delivery, also appear to decrease [11,12].

Moreover, cognitive behavioral therapy and psychological support during fertility treatment have been suggested to result in significantly more viable pregnancies compared with routine care [13-15].

While the evidence supporting the importance of improving and maintaining a healthy lifestyle is abundantly clear, changing habits and behaviors remains difficult [16]. Health-compromising behaviors are hard to stop, and health-promoting behaviors are challenging to adopt, making behavior change particularly difficult. As health care budgets face increasing pressure due to noncommunicable diseases associated with an aging population, workforce shortages, and rising costs of novel medical technologies, it is crucial that lifestyle interventions are cost-effective—for instance, through the use of online programs and apps [17].

Several lifestyle intervention programs for women actively seeking medical support to achieve pregnancy have shown high attrition rates and limited effects [18]. However, mobile health (mHealth) apps have the potential to overcome these obstacles by providing individualized, tailored, and repeated information [19,20]. Indeed, growing evidence suggests that mobile technology can effectively improve inadequate nutrition, lifestyle, and medication adherence [21,22].

Many lifestyle apps are currently available on the market, but very few are specifically designed for couples wishing to have children. To provide guidance for both patients and health care providers (HCPs), the MyFertiCoach (MFC) app was developed as a 6-month integrated lifestyle program with modules on healthy weight; nutrition; exercise; and quitting smoking, alcohol, drugs, and stress.

The MFC app supports couples trying to conceive and pregnant women in modifying unhealthy dietary and lifestyle habits. HCPs can use the app to help patients improve their lifestyle. Based on demographic parameters and an assessment of nutrition and lifestyle habits, a screening process is conducted, and a personalized coaching program consisting of lifestyle modules is created. Interactive elements, visual support, and motivational interviewing are incorporated as much as possible. Additionally, at various points during the program, we will assess whether lifestyle improvements have been achieved across different domains.

This retrospective, observational, single-center evaluation will be the first to assess the impact of the MFC app on changing 7 lifestyle habits in women seeking fertility treatment: healthy weight, diet, exercise, smoking, alcohol, drugs, and stress. Given the established influence of these lifestyle factors on subfertility, we expect that positive changes in these habits will also enhance reproductive health.

## Methods

### Overview

This is a retrospective, single-center evaluation conducted at the Elisabeth-TweeSteden Hospital in Tilburg, the Netherlands. This work does not assess the effectiveness of a new intervention before implementation but rather evaluates an addition to standard care after its integration.

### Ethical Considerations

This study did not undergo formal approval by a medical ethics board for several reasons. First, the app was not purchased for scientific purposes but rather to augment standard care. As such, it received explicit approval from the board of directors for purchase and subsequently became part of standard care.

Additionally, all patients were free to use the app or not; their treatment remained unchanged if they chose not to use it. App usage was entirely voluntary, and data storage complied with national and European GDPR (General Data Protection Regulation) guidelines. The data were anonymized, and none of the researchers had direct access to them. Thus, the hospital purchased an app that was offered to patients as an optional resource. Each patient indicated whether they wanted to use the app and, if so, whether their anonymized data could be used for scientific purposes. As a result, the data set was generated with full patient consent and in compliance with privacy regulations.

Furthermore, our data analysis was conducted retrospectively, long after data collection had been completed. One of the authors (MJ) has previously used a similar approach, analyzing distress thermometer data stored in the electronic patient system [23]. For that study, the institutional review board was consulted, and an exemption was granted as it was classified as file research.

Adding a research paper is not the same as legislation, but the Dutch Central Committee on Research Involving Human Subjects states that “Retrospective research/research with patient files does not fall within the scope of the WMO, as the research subject is not physically involved in the research. Nor have the data that are to be researched been gathered for the sake of the research. The research subjects do not have to change their behaviour for the sake of the research.” [24]. We believe the last sentence is the most relevant: although patients filled out questionnaires and could use the app to support their care and contact their physician, the app did not require them to change their behavior for the sake of care or research. In fact, many patients we approached chose not to participate, and many who did eventually dropped out. They were free to do so. As the data were analyzed retrospectively, we consider our evaluation comparable to file research [25]. Following the decision tree for scientific medical research, our study falls under non-WMO research: it is medical-scientific research, but no patient was required to change procedures or behavior. The app was as freely available as any app that can be purchased from the Apple Store (Apple Inc.) or Google Play (Alphabet Inc.).

### The MyFertiCoach App

The MFC app is a coaching program specifically designed to support lifestyle changes. It was developed by Ferring Pharmaceuticals B.V. in collaboration with a multidisciplinary team of health care professionals, with input from the patients’ association Freya and patients who participated in the test phase.

HCPs can use the app to support patients and their partners while actively monitoring the (pre)active treatment phase. While the patient and their partner are motivated within the domains relevant to them, progress and results are visualized on a dashboard. Data essential for the next steps in treatment can also be used for evaluations and scientific research.

The MFC app promotes lifestyle changes over 6 months through motivation (using motivational interviewing techniques), information (on various domains), and relaxation (by incorporating game elements). It focuses on 7 key domains: achieving a healthy weight, maintaining a healthy diet, engaging in regular exercise, quitting smoking, stopping alcohol consumption, discontinuing drug/anabolic steroid use, and reducing stress through mindfulness. These domains were determined by the evaluation group and are supported by literature demonstrating their influence on the likelihood of a healthy pregnancy.

During the registration process, baseline demographic parameters and questionnaires on dietary and lifestyle habits are completed to screen patients on various factors, including BMI, smoking, nutrition, alcohol use, and stress. These assessments provide insights into patients’ habits and help determine which domains require attention or intervention. The identified domains are then presented to the patient in the order of importance.

Patients were free to choose whether to complete the questionnaires, which took an average of 10 minutes in total. No financial compensation was provided for completing them.

To assess patients’ knowledge, skills, and confidence in managing their own health or illness, both the patient and their partner are asked to complete the Patient Activation Measure questionnaire [26]. This provides HCPs with deeper insights into patients’ perceived ability to improve their health, allowing for adjustments in the level of coaching and motivational interviewing techniques used.

At the first consultation, based on the screening outcomes, the HCP and patient jointly decide which aspects of their lifestyle to focus on. The relevant modules—healthy weight, healthy eating, healthy activity, smoking cessation, alcohol cessation, drug cessation, and stress reduction with mindfulness—are then activated. Once activated, patients receive module-specific questionnaires and general patient-reported outcome measures on a monthly basis. Patients are free to complete these at their convenience, but a baseline screening measure is required for inclusion in the evaluation. The program duration is set at 6 months.

Using the diary page, each patient can access all functionalities that allow them to record their daily mood and receive motivating messages. They can also send messages to their HCP with questions (eg, about nutrition or other lifestyle factors). The HCP can respond at their discretion. Each patient’s progress is visible to the HCP via the MFC platform through domain-specific visualizations. Patients can track their progress using the app.

### Recruitment and Data Collection

All new women aged 18-45 years who visited the fertility clinic were signed up for the MFC app after providing written consent and before their first appointment with the HCP. The MFC program, its modules, and the questionnaires were designed as a screening tool for lifestyle factors and were offered to all new patients as part of standard care.

The data extraction took place on February 1, 2024, and included all patients from January 1, 2022, to December 31, 2023, who completed the screening and had at least one activated module. Patients received a push notification and an email 1 month after the previous questionnaire. Patients who did not provide a baseline score and at least one follow-up score for an outcome (regardless of time) were excluded from further analysis of that particular outcome.

In the case of pregnancy, patients could check a box in the app indicating that they were pregnant (positive test). These patients would then receive additional questionnaires related to their pregnancy. As pregnancy is a defined endpoint, the formal intervention by the app was stopped, meaning these patients were no longer included in the evaluation. However, because some women indicated that they wanted to continue using the app, all modules—except the healthy weight module—remained active for 6 months. The app was never formally stopped by the physician, as it is part of standard care.

### Outcomes

#### Assessing the Impact of the MFC App: Primary and Secondary Measures of Lifestyle Program Benefits

To properly evaluate the MFC app, several outcome measures have been defined to assess any additional benefits of our lifestyle program. The primary outcome was the change in the total risk score (TRS) [10] at the 3- and 6-month follow-ups. The secondary endpoints are changes in BMI, activity score, preconception dietary risk (PDR) score [27], distress score [28], smoking habits, alcohol intake, and program adherence.

#### Total Risk Score

The TRS is based on the Rotterdam Reproduction Risk Score (R3 score), the PDR score, and other existing evidence of associations with reproductive and pregnancy outcomes. An individual’s TRS is defined as the sum of all risk scores per behavior (ie, based on vegetable, fruit, and folic acid intake, as well as smoking and alcohol use after 3 and 6 months) [10].

Vegetable and fruit intake were each subdivided into a risk score of 0, 1, 2, or 3. A score of 0 represents an adequate daily intake (≥200 g of vegetables per day and ≥2 pieces of fruit per day, respectively). Scores of 1 and 2 both indicate a “nearly adequate” intake (vegetable intake of 150 to <200 g and fruit intake of 1.5 to <2 pieces per day), with score 1 reflecting the participant’s intention to change this risk factor and score 2 indicating no such intention. A score of 3 represents an inadequate daily intake (vegetable intake <150 g and fruit intake <1.5 pieces).

If a participant received a score of 1 or 2, an additional question regarding their intrinsic motivation was asked to determine whether they intended to improve their behavior related to this risk factor.

Folic acid supplement use was considered adequate (score 0) or inadequate (score 3) based on whether a participant met the recommendation of taking a 400-mg folic acid supplement daily during the periconceptional period. There is no evidence or recommendation for folic acid supplementation beyond 12 weeks of pregnancy, although this does not mean patients are not allowed to continue its use. Therefore, if a patient became pregnant while using the app, took folic acid for the first 12 weeks of pregnancy, and then stopped, she received a score of 0 for folic acid supplement use. This approach ensured consistency in scoring across all patients, allowing for a more conservative estimate of the app’s benefits.

Risk scores for smoking and alcohol consumption were based on average daily use: no smoking (score 0), smoking 1-5 cigarettes (score 1), 6-14 cigarettes (score 3), or ≥15 cigarettes (score 6); and no drinking (score 0), drinking <1 alcoholic beverage per day (score 1), 1-2 beverages per day (score 2), or ≥2 beverages per day (score 3).

An important distinction is that the TRS is calculated separately for females and their partners (male/female). A higher TRS indicates unhealthier nutrition and lifestyle habits, which lowers the chance of becoming pregnant [10]. Based on previous findings, where a 1-point increase in the TRS was associated with a 21% reduction in pregnancy likelihood, we considered a 1-point decrease in the TRS clinically relevant.

#### Other Scores

We considered a BMI (calculated as weight divided by height squared) between 18.5 and 25 kg/m^2^ to represent a “normal” weight [29]. A BMI ≥29 kg/m^2^ is associated with a lower chance of becoming pregnant, with each unit increase above 29 linked to a 4% decrease in fertility [2]. Therefore, we considered a 1-point drop in the high BMI group clinically relevant.

To determine the activity score, we followed the Dutch physical activity guidelines, which state that adults should be physically active for at least 30 minutes a day on at least five days per week. This activity can include sports, walking, cycling, or strenuous household chores.

To compute a score, we devised 2 sets of 3 questions. The first set assessed the number of active minutes per week (ie, <75, 75 to <150, and ≥150 minutes), while the second set assessed muscle- and bone-enhancing activities (ie, never, once a week, or at least twice a week). Scores ranged from 1 to 3 for each set, with a total possible score between 2 and 6.

The level of distress was measured using the distress score, which provides a general impression of the problems couples experience in 5 areas: practical problems, family/social problems, emotional problems, religious/spiritual problems, and physical problems [30]. Additionally, couples were separately asked to rate their level of distress using a “thermometer” ranging from 0 to 10. The physician could then explore with the couple where their greatest concerns lay and discuss available options to address or reduce any symptoms or concerns as much as possible. A score of 5 or higher on the thermometer indicated a serious “burden.” For our analysis, we only used the thermometer scores.

The PDR score was based on 6 nutritional questions covering the intake of key food groups, in line with dietary recommendations from the Netherlands Nutrition Centre. According to current guidelines, individuals should consume at least four slices of whole wheat bread daily (or an equivalent amount of cereals); use monounsaturated or polyunsaturated oils; and consume ≥200 g of vegetables daily, ≥2 pieces of fruit daily, ≥3 servings of meat or meat substitutes weekly, and ≥1 serving of fish weekly. Patients received 1 point for each recommendation met, with a maximum PDR score of 6, representing highly adequate nutrition according to the Netherlands Nutrition Centre’s guidelines.

The smoking and alcohol scores were based on the number of cigarettes or alcoholic beverages consumed per day. Adherence was measured by the number of active patients over time.

### Statistical Analysis

#### Evaluation Plan

This evaluation was conducted in accordance with a Statistical Analysis Plan (see Multimedia Appendix 1).

#### Descriptives and Transformations

We used descriptive statistics for each outcome of interest: the number of patients and observations for frequency data, and the mean and 95% CI for continuous data. For each outcome, we also calculated the number and percentage of responses per time point, as well as the mean and SD of response time. This allowed us to track response levels at each time point and assess the appropriateness of the predefined time-point categories (eg, 3 months, 6 months).

Some outcomes are known composites of aggregated parameters (eg, BMI), while others are transformations based on the specific manual of the questionnaire. We followed each manual’s guidelines on handling missing data when transforming or aggregating specific questionnaire items.

#### Missing Data

There is no official cut-off for what constitutes an acceptable or unacceptable level of missingness. Additionally, the reason for missing data is often unclear, except in cases where a participant has died. Therefore, we conducted a missing pattern analysis to determine the amount of missing data for each parameter separately and to identify how often multiple parameters are missing simultaneously.

We examined missing patterns across all items in the data set and aimed to classify them into 3 recognized types of missing data patterns: missing completely at random (MCAR), missing at random (MAR), or missing not at random (MNAR) [31]. Of these 3 patterns, MNAR is the most problematic [32,33], as the missingness occurs for a specific reason related to the actual score a participant could have provided but did not.

As there are no formal statistical tests for assessing the size and nature of missing data, we relied heavily on visual inspection using bar charts, box plots, kernel density plots, and matrix plots. For example, if a questionnaire is missing at a particular time point and continues to be missing at subsequent time points, this would appear as a linear increase in missing data. Moreover, if factors such as age or having a partner are likely to influence responses, we would observe different response distributions across groups.

#### Power

As this evaluation was exploratory in nature, we did not conduct a formal power analysis to estimate the sample size required to detect a specific effect size with a given level of statistical significance. Statistical significance was determined by checking whether the 95% CI excluded 0.

#### Statistical Models Used

The main objective of this evaluation is to investigate the effect of following the MFC app program on changes in the TRS after 3 and 6 months. We analyzed the secondary outcomes (ie, BMI, activity score, PDR score, distress score, smoking habits, and alcohol intake) in the same way as the primary outcome, except for program adherence, which was described using descriptive statistics.

To estimate change, our primary analysis focused on patients for whom the intended module was made available. Accordingly, we used the following combinations: activity score for users of the activity module; alcohol score for users of the alcohol module; BMI for users of the weight module; last scores for users of the mindfulness module; the PDR score for users of the nutrition module; and smoking habits for users of the smoking module. The TRSs were analyzed for all participants who responded during the evaluation.

Using a maximum of 7 measurements per patient (1 baseline measurement and 6 follow-up measurements), and in the absence of a clinically relevant cut-off value, we estimated change using a (hierarchical) linear mixed model (LMM) [34]. LMMs are regression models designed to analyze clustered (longitudinal) data and can accommodate both MCAR and MAR data [35].

An important note is that we did not build separate models for the 3- and 6-month time points. As longitudinal data are correlated (ie, multiple measurements from the same patient), we constructed a single model from which the statistical significance of change can be derived at any time point up to the last observed measurement. Using an LMM, we can include time as either a categorical variable (eg, 1 month, 3 months, or 6 months) or as a continuous variable, represented by the number of months or the actual time in days. This approach allows us to evaluate the app’s effect using days rather than months.

Because of the correlated nature of the data, we developed several LMMs with different random effects structures: random intercept, random slope, and random intercept with random slope [36]. This modeling strategy allows us to account for different assumptions, such as patients having the same starting point but different trajectories (random slope), the same trajectory but different starting points (random intercept), or both different starting points and different trajectories (random intercept and random slope).

#### Model Selection

The primary explanatory factors included in the models are time, partner status, age, the number of active modules, and the specific module associated with an outcome (eg, the nutrition module for the PDR score). To appropriately model time as a continuous variable, we will use linear functions, polynomial terms, or natural cubic splines [37].

To evaluate the relevance of these factors, we will iteratively remove parameters from a fully specified fixed-effects model using the chi-square test, considering both main effects and interaction effects. Model accuracy and parsimony will be assessed using the *F* test (analysis of variance) and the Akaike information criterion (AIC), with lower AIC values indicating better model fit. Model assumptions will be evaluated through graphical diagnostics and visualizations.

It is important to note that we will not build separate models for the 3- and 6-month time points. Instead, we will construct a single model from which the statistical significance of change can be derived at any time point up to the last observed measurement.

The analysis of the primary and secondary outcomes will result in several different models. This raises concerns about spurious results, as each model carries a 5% false-positive rate when determining statistical significance. To safeguard against such statistically spurious findings, it is important to assess whether the 7 outcomes are correlated at any given time point.

To address this, we will first create a correlation matrix using Spearman rank correlation, as many of the outcomes are ordinal in nature. If correlations are consistently higher than 0.50 between outcomes and across time points, we will include the correlated outcomes as predictors in the original LMM models. For example, if there is a correlation between distress and BMI, the distress outcome model will include both time and BMI as predictors.

As all variables have a clinical connection to the outcomes of interest, we allowed this form of statistical reasoning to guide variable inclusion. However, the final decision was based on model estimates for each variable, taking into account that multicollinearity can inflate SEs and affect variance estimates in mixed models. In summary, we included variables deemed both clinically important and statistically necessary.

#### Multiple Imputations in the Case of MNAR

Although patients may exhibit intermittent missingness—commonly associated with MAR situations and addressed using MAR models or multiple imputations—a sudden stop (ie, monotone missingness or drop out) strongly indicates an MNAR response. This is problematic for our analysis if it occurs before the 3- or 6-month follow-up. To address this, we will create a joint model, which combines an LMM with a survival model [38,39]. This approach is recognized as a viable alternative to older methods such as pattern-mixture modeling [40]. One of the authors has previously applied this method successfully to analyze quality-of-life changes in a population of patients with palliative pancreatic cancer [41].

The joint model yields 2 key outputs: (1) a survival model on program adherence, which incorporates information from the LMM; and (2) an LMM that adjusts its estimates based on the survival model. For each joint model, we used Markov chain Monte Carlo with 4 chains and 100,000 iterations, of which 10,000 were designated as burn-in. Model fit was assessed using trace and density plots.

### Sensitivity Analyses

#### Adjusting for Timing Discrepancies in Longitudinal Data: Realignment of Assessment Periods

When analyzing longitudinal data, there is often a discrepancy between the intended timing of questionnaire returns and the actual return dates. This means that a 3-month assessment of an intervention may, in practice, represent a time frame that is either longer or shorter than planned. To address this, we created new time categories that better align with the intended schedule of questionnaire returns. For example, T3 is defined as 90 days plus or minus 14 days, and T6 as 180 days plus or minus 14 days.

#### Statistical Package Used

All analyses were performed using R Statistical Software (v4.1.2; R Foundation), a widely recognized and accepted program for statistical analysis. For the LMMs, we used the lme4 package. For the joint models, we used the JMbayes2 package in conjunction with the nlme package for constructing the LMM component. The survival package was used to build the Cox proportional hazards component of the joint models.

## Results

### Patient Characteristics

Table 1 presents the response rates during the evaluation period, along with response times, which varied by questionnaire (Figure S4 in Multimedia Appendix 2) and over time (Figure S2 in Multimedia Appendix 2). In total, 1815 patients were invited to participate, and all of them agreed to join, achieving a 100% recruitment rate. Of these, 818 patients (45.07%) agreed to participate. The mean age of the invited population was 34.1 (SD 5.46) years. A comparison between responders and nonresponders showed similar age distributions: 33.0 (SD 5.03) years for responders versus 35.1 (SD 5.63) years for nonresponders (Figure S1 in Multimedia Appendix 2).

**Table 1:** Number and percentage of responses, and the mean and SD of the response time.

Questionnaire and condition						Measurement moments																
						Screening		T0		T1		T2		T3		T4		T5		T6		
**Total risk score**																						
	**Regardless of the active module**																					
		n (%)			1815 (100)		546 (30.1)		270 (14.9)		148 (8.2)		91 (5.0)		50 (2.8)		36 (2.0)		21 (1.2)			
		Mean (SD)			N/A^a^		N/A		N/A		N/A		N/A		N/A		N/A		N/A			
**Activity**																						
	**Regardless of** **the** **active module**																					
				n (%)	1815 (100)		758 (41.8)		235 (12.9)		139 (7.7)		85 (4.7)		50 (2.7)		34 (1.9)		20 (1.1)			
				Mean (SD)	N/A		0 (0)		55.6 (40.9)		89.6 (25.1)		120.6 (23.5)		154.1 (26.3)		186.3 (24.0)		220.3 (27.8)			
	**Only active module exercise**																					
			n (%)		444 (100)		438 (98.6)		233 (52.5)		138 (31.1)		85 (19.1)		50 (11.3)		34 (7.7)		20 (4.5)			
			Mean (SD)		N/A		0 (0)		55.8 (41.0)		89.7 (25.1)		120.6 (23.5)		154.1 (26.3)		186.3 (24.0)		220.6 (27.8)			
**Alcohol**																						
	**Regardless of the active module**																					
			n (%)		1815 (100)		761 (41.9)		210 (11.6)		113 (6.2)		61 (3.4)		38 (2.1)		23 (1.3)		7 (0.4)			
			Mean (SD)		N/A		0 (0)		34.4 (23.7)		62.0 (24.0)		92.2 (26.2)		130.9 (31.9)		158.6 (31.2)		196.3 (45.6)			
	**Only active module alcohol**																					
			n (%)		209 (100)		199 (95.2)		95 (45.5)		42 (20.1)		26 (12.4)		14 (6.7)		11 (5.3)		5 (2.4)			
			Mean (SD)		N/A		0 (0)		46.2 27.2)		76.2 (27.1)		104.3 (28.4)		154.5 (38.4)		177.4 (35.2)		211.8 (45.5)			
**BMI (kg/m** ^**2**^ **)**																						
	**Regardless of the active module**																					
			n (%)		1815 (100)		792 (43.6)		209 (11.5)		115 (6.3)		73 (4.0)		45 (2.5)		32 (1.8)		19 (1.0)			
			Mean (SD)		N/A		0 (0)		57.9 (43.5)		93.3 (30.1)		125.9 (27.7)		154 (25.2)		187.1 (25.3)		225.8 (27.6)			
	**Only active module weight**																					
			n (%)		420 (100)		416 (99)		207 (49.3)		115 (27.4)		73 (17.4)		45 (10.7)		32 (7.6)		19 (4.5)			
			Mean (SD)		N/A		0 (0)		57.9 (43.5)		93.3 (30.1)		125.9 (27.7)		154.0 (25.2)		187.1 (25.3)		225.8 (27.6)			
**Distress**																						
	**Regardless of the active module**																					
			n (%)		1815 (100)		764 (42.1)		84 (4.6)		42 (2.3)		27 (1.5)		17 (0.9)		13 (0.7)		7 (0.4)			
			Mean (SD)		N/A		0 (0)		65.7 (64.5)		115.5 (89.9)		160.8 (110.3)		203.8 (135.5)		220.0 (108.2)		223.9 (28.7)			
	**Only active module mindfulness**																					
			n (%)		157 (100)		153 (97.5)		83 (52.9)		42 (26.8)		27 (17.2)		17 (10.8)		13 (8.3)		7 (4.5)			
			Mean (SD)		N/A		0 (0)		66.4 (64.5)		115.5 (89.9)		160.8 (110.3)		203.8 (135.5)		220.0 (108.2)		223.9 (28.7)			
**Preconception dietary risk**																						
	**Regardless of the active module**																					
			n (%)		1815 (100)		783 (43.1)		380 (20.9)		218 (12.0)		131 (7.2)		80 (4.4)		56 (3.1)		32 (1.8)			
			Mean (SD)		N/A		0 (0)		55.2 (42.3)		87.8 (34.6)		123 (42.5)		155.7 (50.2)		192.6 (58.1)		222.9 (26.9)			
	**Only active module nutrition**																					
			n (%)		693 (100)		680 (98.1)		379 (54.7)		218 (31.5)		131 (18.9)		80 (11.5)		56 (8.1)		32 (4.6)			
			Mean (SD)		N/A		0 (0)		55.3 (42.3)		87.8 (34.6)		123 (42.5)		155.7 (50.2)		192.6 (58.1)		222.9 (26.9)			
**Smoking**																						
	**Regardless of the active module**																					
			n (%)		1815 (100)		761 (41.9)		579 (31.9)		375 (20.7)		262 (14.4)		191 (10.5)		131 (7.2)		94 (5.2)			
			Mean (SD)		N/A		0 (0)		20 (20.4)		44.4 (24.5)		71.7 (32.6)		88.6 (40.0)		114.1 (41.9)		129.6 (51.6)			
	**Only active module smoking**																					
			n (%)		130 (100)		119 (91.5)		82 (63.1)		53 (40.8)		42 (32.3)		28 (21.5)		18 (13.8)		11 (8.5)			
			Mean (SD)		N/A		0 (0)		16.4 (16.5)		41.4 (20.5)		63.9 (29.6)		85.0 (34.8)		105.8 (33.2)		126.2 (40.5)			

^a^N/A: not applicable.

Among the 818 participants, 532 (65%) also included their partner. The partner-to-no-partner ratio differed significantly between those who accepted the invitation (532/818, 65.0% vs 286/818, 35.0%) and those who did not (323/997, 32.4% vs 674/997,67.6%; *χ*^2^_1_=190.81; *P*<.001).

When age distribution was further stratified by partner status, the results remained consistent. For nonparticipants, the mean ages were 34.0 (SD 4.59) years with a partner and 35.6 (SD 6.02) years without a partner. For participants, the mean ages were 32.1 (SD 4.22) years with a partner and 34.7 (SD 4.22) years without a partner.

Ultimately, data from 798 of 1815 patients (43.97% of those invited) were included in the analysis. These participants had a mean age of 33.0 (SD 5.04) years, with 517 (64.8%) including their partner. Of the 784 (98.2%) participants who reported their educational background, the majority (423/784, 53.9%) held a higher professional or university degree. Notably, higher education levels were associated with greater follow-up rates (see Figure S5 in Multimedia Appendix 2).

### MFC Programs and Grading by Patients

Patients engaged with a diverse range of personal programs. Specifically, 693 patients included a nutrition module, while 130 patients included a smoking module. In total, the distribution of active modules per patient was as follows: we included 113 patients with 1 active module, 271 patients with 2 active modules, and 289 with 3 active modules; 4, 5, and 6 active modules were attached to 89, 33, and 3 patients, respectively. When asked to grade the MFC app on the seventh and fourteenth day of using the app (Figure S6 in Multimedia Appendix 2), 115 and 68 patients, respectively, gave a median score of 7 (IQR 3).

### Patient Response and Response Time

The percentage of patients who filled out a questionnaire dropped rapidly across time (Figure S9 in Multimedia Appendix 2). The percentage of patients who had both a baseline score and at least one follow-up score (Figure S11 in Multimedia Appendix 2) ranged from 84 out of 764 (11.0%) patients for the distress score to 579 out of 761 (76.1%) patients for the smoking score, regardless of the module included. Across questionnaires, we received an average follow-up response rate of 38.3% (average of 282 patients/average of 737 patients). Compared with patients who did not return a follow-up questionnaire (see Figures S12-S14 and S16 in Multimedia Appendix 2), those who did respond had different median baseline scores for BMI (29.4 vs 24.3 kg/m^2^), exercise (3 vs 5), distress score (6 vs 2), and alcohol (2 vs 0.1). However, the median scores for the TRS (3), PDR (4), and smoking (0) did not differ at follow-up. The follow-up percentage did differ much between those who included a partner and those who did not (see Figure S8 in Multimedia Appendix 2). When we accounted for the number of active modules, the graphs showed a slightly different pattern (see Figures S7, S8, and S10 in Multimedia Appendix 2), but the data became too sparse for additional statistical analysis. At baseline, the response rate ranged from 41.76% (758/1815) to 43.64% (792/1815), regardless of the active module, and from 91.5% (119/130) to 99.0% (416/420) per active module. These percentages declined over time, reaching 8.5% (11/130) for the smoking module and 2.4% (5/209) for the alcohol module at the last time point (see Figure S15 in Multimedia Appendix 2). The average response time did not align with the expected response time. While all patients completed their baseline questionnaire on time, the average response time for the first follow-up, regardless of the module, ranged from 20 days (smoking score) to 65.7 days (distress score). When considering only patients with active modules, the response times were 16.4 and 66.4 days for the smoking and distress scores, respectively.

As time progressed, the gap between actual and expected response times widened. At the fifth time point—approximately 5 months after the follow-up—response times, regardless of the module, ranged from 220 days for the distress score to 114 days for the smoking score. The longest response time was observed for BMI, with an average of 226 days for the 6-month follow-up.

### Sensitivity Analyses

Although secondary outcomes showed correlations at each time point, none consistently exceeded 0.5 (see Figures S24 and S25 in Multimedia Appendix 2). Therefore, we did not use any secondary outcomes to explain each other.

When we created new time categories that better aligned with the expected return of questionnaires (eg, T3 was defined as 90 days plus minus 14 days, T6 as 180 days plus minus 14 days), we reduced our effective sample size (see Figure S3 in Multimedia Appendix 2). However, this did not alter the distribution of values across the outcomes (see Figure S14 in Multimedia Appendix 2). Consequently, we used the Tx categories for our LMM models (main analysis) while using the actual time until questionnaire submission for our joint models, which also accounted for missing data.

### Missing Data Pattern Analysis

The missing data pattern analysis revealed a monotone pattern (see Figures S31-S36 in Multimedia Appendix 2), meaning that once a patient stopped returning questionnaires, they dropped out entirely. This suggests a potential MNAR pattern. Consequently, we successfully fitted joint models for the TRS (see Figure S19 in Multimedia Appendix 2), activity (see Figure S26 in Multimedia Appendix 2), alcohol intake (see Figure S27 in Multimedia Appendix 2), BMI (see Figure S29 in Multimedia Appendix 2), PDR (see Figure S30 in Multimedia Appendix 2), and smoking habits (see Figure S28 in Multimedia Appendix 2). The results from the joint models align with the outcomes of the LMM models, although they are somewhat harder to interpret due to the inclusion of splines. Therefore, we reported the output of the LMM models, as they better followed the original questionnaire sampling. A joint model for distress did not converge. In summary, the MNAR analysis via joint modeling did not warrant further investigation.

### Results for the Primary and Secondary Outcomes

The model selection results (see Figures S17-S23 in Multimedia Appendix 2) show 14 models for alcohol intake, BMI, activity, and the distress score. For PDR and smoking habits, we created 15 models, while for the TRS, we included 13 different models. In almost all selection procedures, the AIC was lowest for models that included a main effect of time (as a dummy variable), partner, age, and the number of active modules.

**Figure 1:**
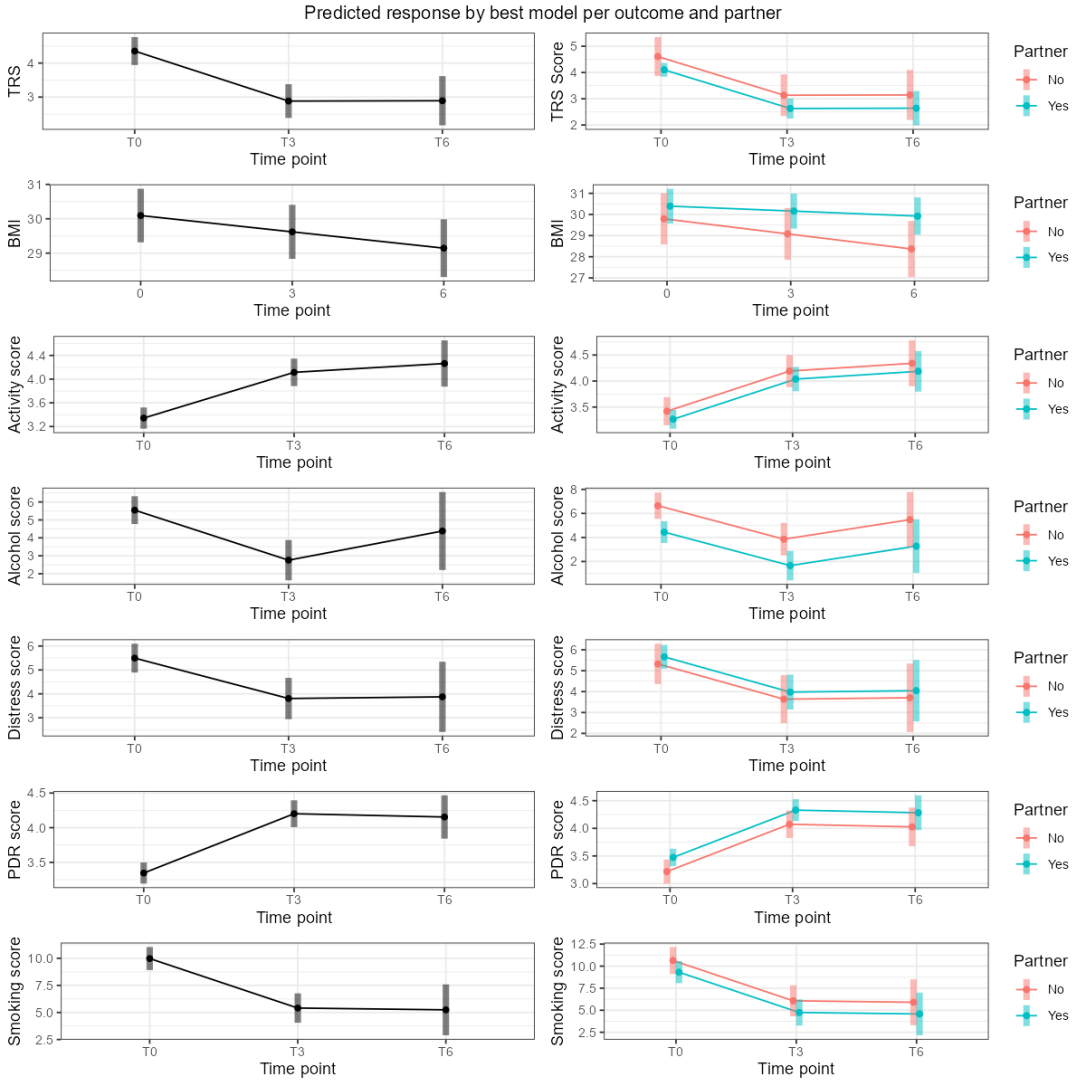
Predicted response by best model per outcome and partner.

Figure 1 and Table 2 show the results for primary and secondary outcomes at baseline, 3-month, and 6-month follow-ups. The number of patients and observations included ranged from 424 patients (1040 observations) for the TRS to 83 patients (272 observations) for the distress score.

**Table 2:** Results following the MFC^a^ app at the 3- and 6-month follow-up.^b^

Questionnaire	Values		Measurement moments												
			T0		T3					T6					
	N	Observed	Mean	95% CI	Mean	95% CI	Δ^c^	*P* value	Mean		95% CI	Δ	*P* value		
Total risk score	424	1040	3.81	3.43-4.19	2.34	1.87-2.81	–1.47	<.001	2.35		1.65-3.06	–1.46	<.001		
Activity	230	786	3.41	3.25-3.57	4.18	3.97-4.40	0.77	<.001	4.33		3.95-4.7	0.92	<.001		
Alcohol	94	286	5.43	4.71-6.14	2.64	1.55-3.72	–2.79	<.001	4.26		2.10-6.42	–1.16	.53		
BMI (kg/m^2^)	207	698	30.1	29.4-30.8	29.6	28.9-30.3	–0.47	<.001	29.1		28.4-29.9	–0.95	<.001		
Distress	83	272	5.53	4.94-6.12	3.84	2.99-4.70	–1.69	<.001	3.91		2.45-5.37	–1.62	.07		
Preconception dietary risk	265	375	3.42	3.30-3.55	4.28	4.11-4.45	0.86	<.001	4.23		3.93-4.53	0.81	<.001		
Smoking	313	81	9.95	8.89-11.02	5.38	4.03-6.75	–4.75	<.001	5.22		2.87-7.56	–4.74	<.001		

^a^MFC: MyFertiCoach.

^b^Results are based on the most optimal model following an extensive model selection procedure.

^c^Δ: difference between categories.

For all outcomes, the change in score compared with baseline was statistically significant at 3 months (at the 5% level or *P*<.001) and considered positive (eg, lower BMI, reduced smoking, or improved nutrition). These changes remained statistically significant (*P*<.001)at 6 months, except for alcohol intake (*P*<.53) and distress levels (*P*<.07). For alcohol, part of the initial positive response (Δ –2.79; *P*<.001) diminished by 6 months (Δ –1.16; *P*<.531). For distress, the initial statistically significant change (Δ –1.69; *P*<.001) shifted to a positive trend (Δ –1.62; *P*<.073). BMI was the only outcome that continued improving over 6 months (30.1 vs 29.6 vs 29.1 kg/m^2^; *P*<.001). For the TRS, the changes were clinically relevant at both 3 and 6 months. For BMI, clinical relevance was only observed at 6 months.

When we stratified the longitudinal analysis by partner presence (Figure 1 and Table 3), statistical significance was maintained at 3 months (*P*<.001), except for BMI, which was no longer significant in the group that included a partner (30.4 vs 30.2 kg/m^2^ vs 29.9 kg/m^2^; *P*<.07). However, the group without a partner showed an even greater decrease in BMI (29.8 vs 29.1 vs 28.4 kg/m^2^; *P*<.001). Regardless of partner status, the change in alcohol intake remained nonsignificant (*P*=.53).

**Table 3:** Results following the MFC^a^ app at the 3- and 6-month follow-up.^b^

Questionnaire	Partner	Values			Measurement moments											
					T0			T3					T6			
		N	Observed	Mean		95% CI	Mean		95% CI	Δ^c^	*P* value	Mean		95% CI	Δ	*P* value
**Total risk score**		424	1040													
	Yes			3.56		3.35-3.77	2.1		1.74-2.44	1.47	<.001	2.1		1.47-2.73	1.46	<.001
	No			4.07		3.34-4.79	2.59		1.82-3.37	1.47	<.001	2.6		1.67-3.54	1.46	<.001
**Activity**		230	786													
	Yes			3.33		3.17-3.50	4.1		3.89-4.33	–0.77	<.001	4.26		3.87-4.64	–0.9	<.001
	No			3.49		3.23-3.75	4.26		3.96-4.56	–0.77	<.001	4.41		3.98-4.84	–0.9	<.001
**Alcohol**		94	286													
	Yes			4.33		3.44-5.21	1.5		0.32-2.75	2.79	<.001	3.2		0.93-5.39	1.2	.53
	No			6.52		5.51-7.54	3.7		2.44-5.03	2.79	<.001	5.4		3.09-7.63	1.2	.53
**BMI (kg/m^2^)**		207	698													
	Yes			30.4		29.6-31.2	30.2		29.3-31.0	–0.23	.07	29.9		29.0-31.0	–0.5	.07
	No			29.8		28.6-31.0	29.1		27.9-30.3	–0.71	<.001	28.4		27.0-29.7	–1.4	<.001
**Distress**		83	272													
	Yes			5.70		5.14-6.26	4.01		3.18-4.84	1.69	<.001	4.08		2.62-5.54	1.62	.07
	No			5.36		4.40-6.32	3.67		2.53-4.82	1.69	<.001	3.74		2.11-5.37	1.62	.07
**Preconception dietary risk**		1265	375													
	Yes			3.55		3.42-3.69	4.41		4.23-4.59	–0.86	<.001	4.36		4.06-4.66	–0.81	<.001
	No			3.30		3.10-3.49	4.15		3.92-4.38	–0.86	<.001	4.10		3.77-4.44	–0.81	<.001
**Smoking**		313	81													
	Yes			9.29		8.03-10.54	4.72		3.23-6.20	–4.75	<.001	4.55		2.13-6.97	–4.74	<.001
	No			10.64		9.09-12.14	6.04		4.29-7.80	–4.75	<.001	5.88		3.28-8.47	–4.74	<.001

^a^MFC: MyFertiCoach.

^b^Results are based on the most optimal model following an extensive model selection procedure.

^c^Δ: difference between categories.

When we further analyzed outcomes by partner presence and time point (Table 4), we observed lower alcohol scores (Δ –2.2; *P*<.001) and higher PDR scores (Δ 0.256; *P*=.03) in patients who included their partner. These were both main effects. However, for BMI, we identified an interaction effect, showing a more pronounced decline at 6 months (Δ –1.555; *P*=.04) in patients who did not include their partner.

**Table 4:** Results following the MFC^a^ app at the 3- and 6-month follow-up^b^.

Questionnaire and partner			Value					Measurement moments																		
								T0							T3							T6				
			N		Observed		Mean			Δ^c^ 95% CI		Δ *P* value		Mean			Δ 95% CI		Δ *P* value		Mean			Δ 95% CI		*P* value
**Total risk score**			424		1040																					
	Yes					3.56			0.505 (–0.244 to 1.25)		.19		2.1			0.505 (–0.244 to 1.25)		.19		2.1			0.505 (–0.244 to 1.25)		.19	
	No					4.07			N/A^d^		N/A		2.59			N/A		N/A		2.6			N/A		N/A	
**Activity**			230		786																					
	Yes					3.33			0.154 (–0.135 to 1.051)		.29		4.1			0.154 (–0.135 to 1.051)		.29		4.3			0.154 (–0.135 to 1.051)		.29	
	No					3.49			N/A		N/A		4.3			N/A		N/A		4.4			N/A		N/A	
**Alcohol**			94		286																					
	Yes					4.33			2.2 (0.922 to 3.47)		<.001		1.5			2.2 (0.922 to 3.47)		<.001		3.2			2.2 (0.922 to 3.47)		<.001	
	No					6.52			N/A		N/A		3.7			N/A		N/A		5.4			N/A		N/A	
**BMI (kg/m^2^)**			207		698																					
	Yes					30.4			–0.599 (–1.96 to 0.767)		.39		30.2			–1.077 (–2.46 to 0.3021)		.12		29.9			–1.555 (–3.06 to –0.0518)		.04	
	No					29.8			N/A		N/A		29.1			N/A		N/A		28.4			N/A		N/A	
**Distress**			83		272																					
	Yes					5.70			–0.339 (–1.37 to 0.691)		.51		4.01			–0.339 (–1.37 to 0.691)		.51		4.08			–0.339 (–1.37 to 0.691)		.51	
	No					5.36			N/A		N/A		3.67			N/A		N/A		3.74			N/A		N/A	
**Preconception dietary risk**			1265		375																					
	Yes					3.55			–0.256 (–0.484 to –0.028)		.03		4.41			–0.256 (–0.484 to –0.028)		.03		4.36			–0.256 (–0.484 to –0.028)		.03	
	No					3.30			N/A		N/A		4.15			N/A		N/A		4.10			N/A		N/A	
**Smoking**			313		81																					
	Yes					9.29			–0.909 (–0.485 to 1.458)		.15		4.72			–0.909 (–0.485 to 1.458)		.15		4.55			–0.909 (–0.485 to 1.458)		.15	
	No					10.64			N/A		N/A		6.04			N/A		N/A		5.88			N/A		N/A	

^a^MFC: MyFertiCoach.

^b^Results are based on the most optimal model following an extensive model selection procedure.

^c^Δ: difference between categories.

^d^N/A: not applicable.

## Discussion

### Principal Findings

Our findings indicate that introducing a lifestyle app can immediately improve several lifestyle factors associated with subfertility and the success of fertility treatment. Specifically, the assessment of the TRS shows that app usage leads to a clinically relevant improvement. The peak effect of the intervention appears to occur around the third month, after which improvements plateau—except for BMI, which remained clinically relevant at 6 months. The MFC app was designed with input from both patients and health care professionals to ensure it targeted lifestyle domains deemed most useful. As a result, the app was intended to be more than just a digital summary of suboptimal lifestyle behaviors. Through shared decision-making, both patients and HCPs contributed to the final selection of active modules, integrating the app into the broader treatment plan. From a preventive medicine perspective, the app provided a valuable starting point and contributed to more efficient care, offering an inexpensive way to influence lifestyle. This impact is also reflected in the data, where most effects were observed immediately after app use began.

However, it appears that not everyone is eager to start an intervention of this type. While we cannot determine with certainty why some individuals chose not to participate, we observed lower participation rates among those with lower levels of education and those without a partner. It remains unclear whether the presence of a partner played a significant role in the acceptance of this evaluation. However, even when better follow-up led to a more favorable effect of the app, partner participation did not consistently enhance its effectiveness.

On an individual level, the reasons why some patients stopped using the app—or never started—remain uncertain. One plausible explanation is that certain users did not identify with the app, despite extensive screening and discussions. Another possibility is that aspects of the app’s design or usability did not appeal to everyone, although we do not have specific data to confirm this.

In summary, we believe the MFC app is a cost-effective addition to standard care, helping subfertile women and their partners implement necessary lifestyle changes. Financial costs are minimal, and health care professionals require little time investment. While the app primarily attracts a specific group—those with higher education and a partner—it does lead to meaningful improvements in key lifestyle factors.

### Limitations

Because of the nature of our evaluation, we were unable to account for all known and unknown confounders. Despite conducting several sensitivity analyses, we cannot rule out the possibility that the temporal differences—or lack thereof—observed between patients, including those with a partner, may be influenced by factors beyond patient characteristics or the app itself. Additionally, the pattern of missing data cannot be formally assessed in a quantitative manner, meaning we do not know why patients dropped out when they did.

Furthermore, we did not include a formal comparative group, so we cannot determine how outcomes would have developed in patients who did not receive guidance through the app. These limitations—along with others, such as the lack of randomization or blinding—prevent us from drawing causal inferences.

Many uncertainties remain. For example, we do not know why patients dropped out, nor do we have full transparency regarding the exact selection process. Although modules were chosen through shared decision-making, it is unclear whether the final choice was driven solely by motivation. However, this does not imply that a lack of improvement is due to low motivation or suboptimal care. It is possible that the interventions themselves did not sufficiently support patients in improving suboptimal lifestyle factors.

Special consideration should be given to folic acid use and pregnancy. While we did not extract these data, it is possible that some patients became pregnant while using the app and continued to use it. As Dutch guidelines recommend folic acid supplementation during the first 3 months of pregnancy, some patients may not have used folic acid, leading to a higher TRS. If this is the case, the app’s beneficial effect could be even greater than our current estimates suggest.

The consistency of findings across LMMs and joint models does not eliminate the potential limitations of our study. While LMMs can handle data that are MAR and joint models can address missing-not-at-random (MNAR) data by incorporating estimates from survival analysis, no statistical safeguard can fully account for the high amount of missing data in our evaluation. Ultimately, there is no way to account for what we do not know.

As a result, we cannot claim that the high probability of MNAR data was entirely mitigated by the joint models. However, we can state that incorporating survival models alongside mixed models produced estimates that were very similar to those obtained using mixed models alone. This strengthens our confidence in the robustness of the model estimates given the available data. Nonetheless, our findings should be interpreted with caution due to the high level of missing data—an inherent limitation when evaluating interventions in standard care settings.

Additionally, our findings are based on a select group of patients from a single center in the Netherlands, where we observed a high rate of attrition during implementation. As we cannot fully determine the reasons for dropout, there is a potential risk of bias, as those who continued participating may differ systematically from those who dropped out.

### Comparison With Prior Work

In the Netherlands, 2 other groups are actively developing apps to support treatment: the international LIFESTYLE study, in which the Medical University of Groningen participates [6,7,42], and the Smarter Pregnancy platform at Erasmus Medical Center [43]. However, these initiatives primarily involve hands-on interventions, making them financially expensive and time-intensive, although they tend to yield better and more stable responses. This suggests that maintaining patient motivation may require a similar level of investment from health care professionals.

Lifestyle changes are notoriously difficult to achieve and sustain. Eating, drinking, and smoking are habits that provide immediate gratification, while their negative effects manifest only in the long term. Psychological stressors are also challenging to address and vary depending on their nature—for example, losing a loved one differs from facing persistent financial difficulties. Consequently, our findings of immediate but not long-lasting changes align with what has been extensively documented in previous research.

Patients varied significantly in their personal trajectories. Some showed substantial improvement, others experienced only marginal benefits, and some saw no measurable change despite their efforts. Analyzing heterogeneous data often results in marginal main effects which, even when statistically significant, may not translate into meaningful clinical change. Attempts to include interaction effects—such as partner presence, number of active modules, and age—did not provide clearer insights into why some patients responded well while others did not.

However, given the nature of the app and the study setting, the observed dropout rates were not unexpected. We aimed to facilitate lifestyle changes in a predominantly working-class population with limited health literacy. This does not imply that these patients cannot benefit from or are unwilling to engage with such interventions, but rather that additional efforts are necessary to achieve long-term change. Therefore, evaluations of mHealth apps should not rely solely on questionnaires but should also incorporate in-depth follow-up interviews, which we plan to conduct in future development stages.

### Conclusions

Our evaluation of an mHealth app in standard care, designed to support women seeking pregnancy, demonstrated an immediate and clinically relevant improvement in key lifestyle parameters. Further research is needed to identify causal pathways leading to sustained effectiveness. To maintain and enhance these results, patient-specific programs must be further tailored.

## Appendix

Multimedia Appendix 1 Supplementary figures.

Multimedia Appendix 2 Statistical analysis plan.

## Abbreviations

AIC: Akaike information criterion
GDPR: General Data Protection Regulation
HCP: health care provider
LMM: linear mixed model
MAR: missing at random
MCAR: missing completely at random
MFC: MyFertiCoach
mHealth: mobile health
MNAR: missing not at random
PDR: preconception dietary risk
TRS: total risk score

## Data Availability

The data sets generated during or analyzed during this evaluation are not publicly available due to privacy reasons. However, because the patients give explicit consent that their data can be anonymously used for scientific research, the data are available from the corresponding author upon reasonable request.
