# Supplementary figures and images for: Evaluation of the MyFertiCoach Lifestyle App for Subfertile Couples: Single-Center Evaluation of Augmented Standard Care
Source: JMIR Form Res. 2025 Mar 10;9:e64239. doi: 10.2196/64239 (PMC11933746; doi:10.2196/64239)

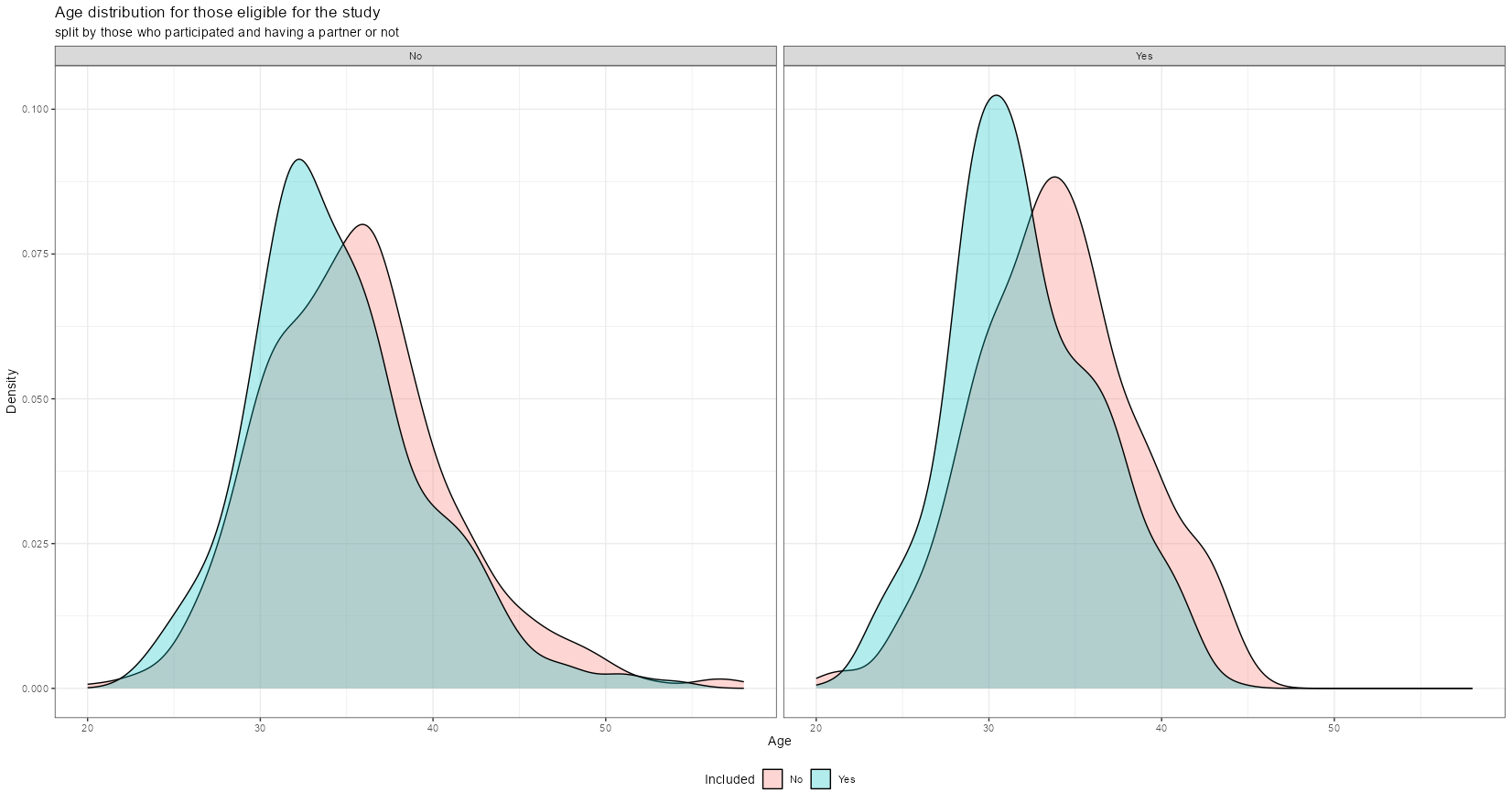

Supplement: Multimedia Appendix 1 [file formative_v9i1e64239_app1.zip › S1 Age distribution for those eligible for the study.png]

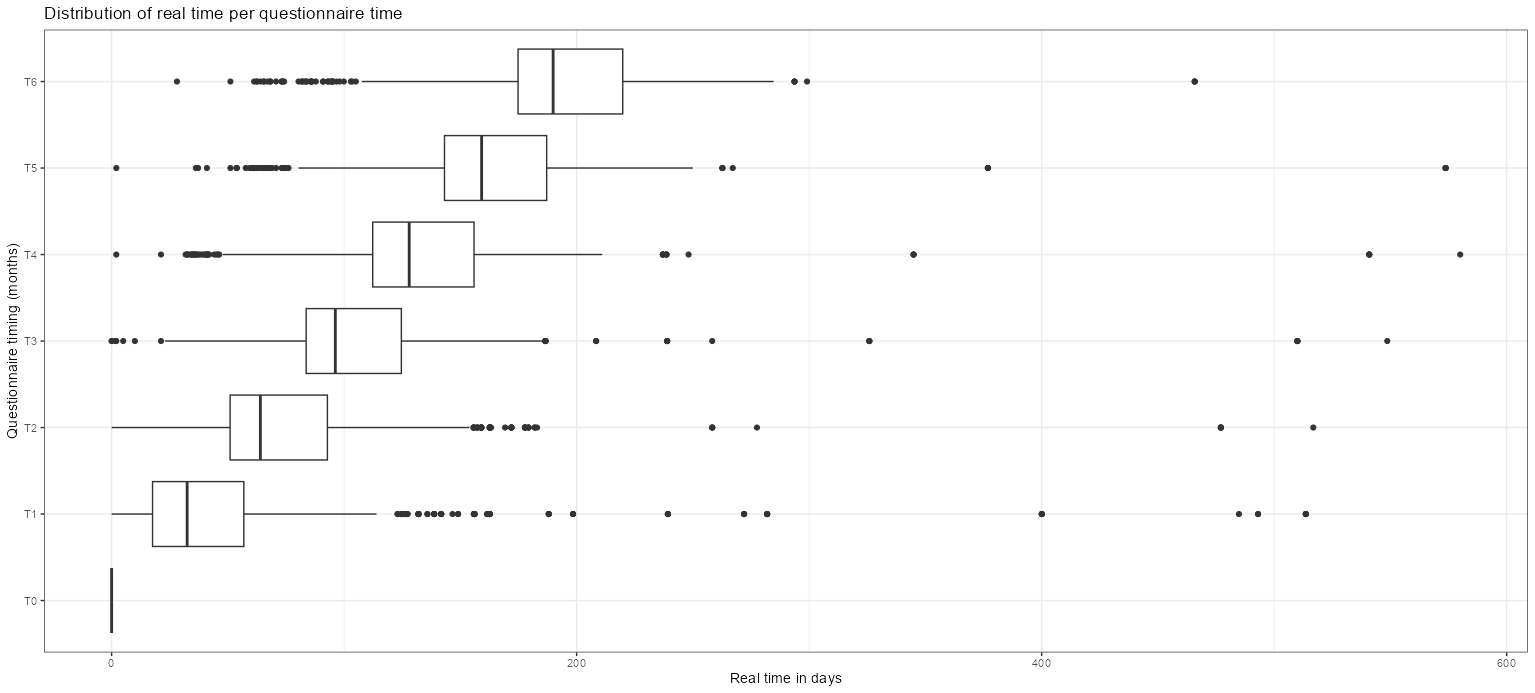

Supplement: Multimedia Appendix 1 [file formative_v9i1e64239_app1.zip › S2 Distirbution of real time per questionnaire time.png]

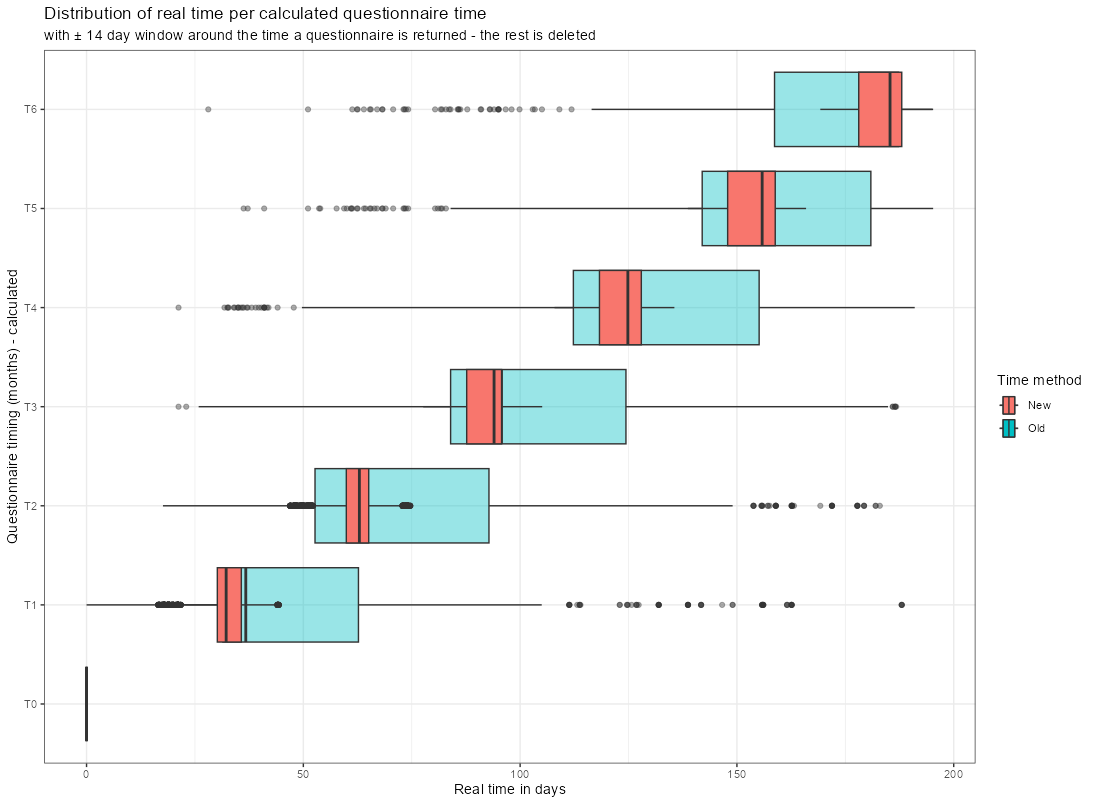

Supplement: Multimedia Appendix 1 [file formative_v9i1e64239_app1.zip › S3 Distribution of real time per calculated questionnaire time.png]

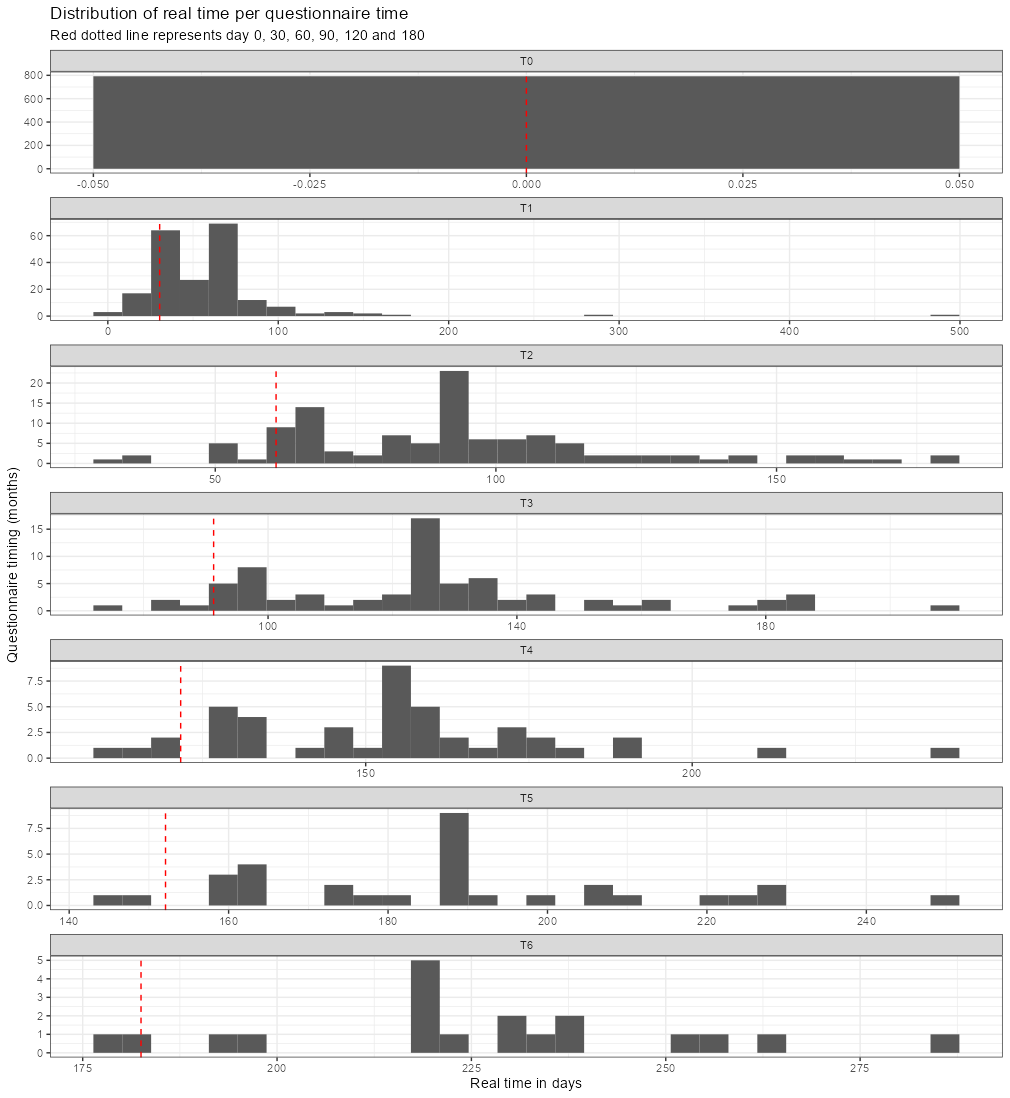

Supplement: Multimedia Appendix 1 [file formative_v9i1e64239_app1.zip › S4 Distribution of real time per questionnaire time.png]

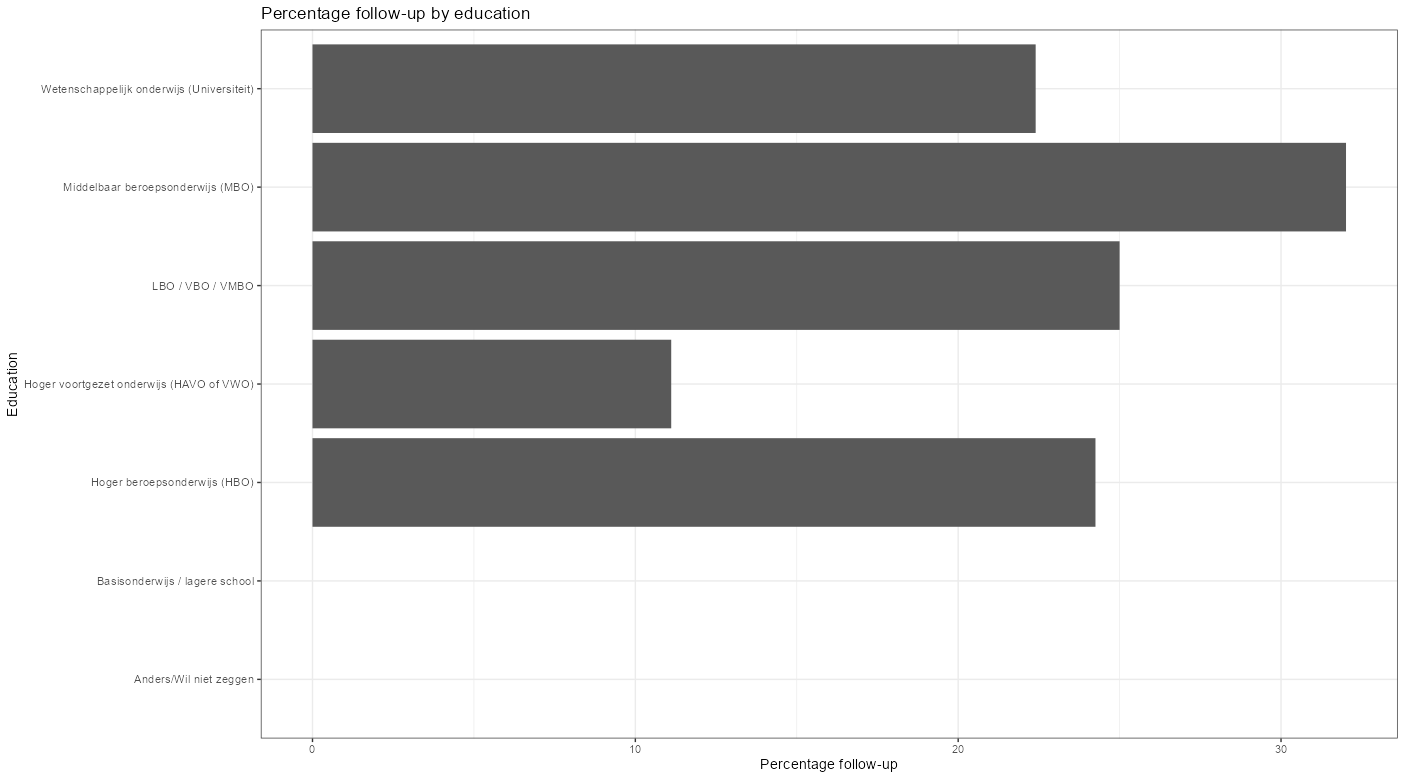

Supplement: Multimedia Appendix 1 [file formative_v9i1e64239_app1.zip › S5 Followup by education.png]

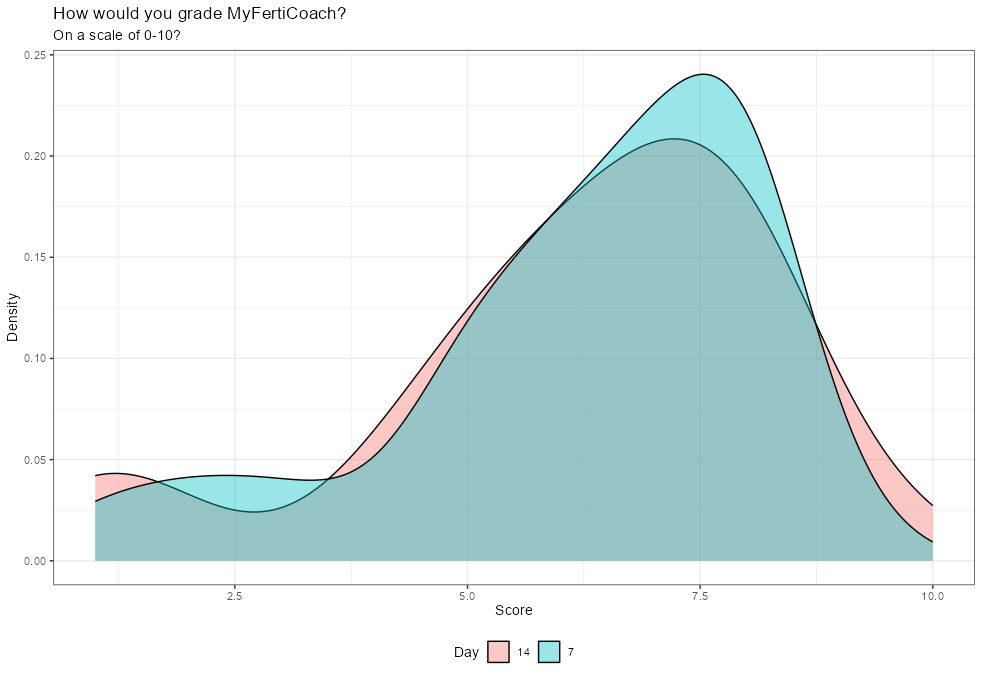

Supplement: Multimedia Appendix 1 [file formative_v9i1e64239_app1.zip › S6 How would you grade MyFertiCoach.png]

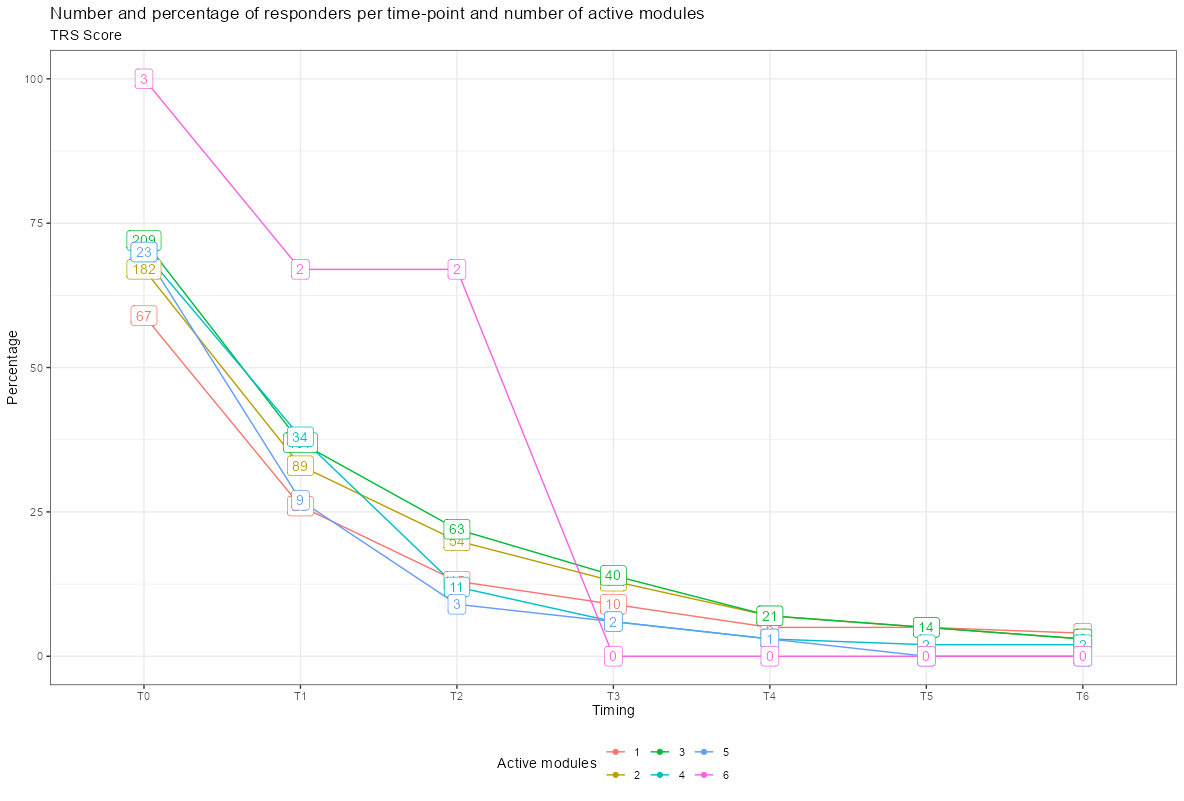

Supplement: Multimedia Appendix 1 [file formative_v9i1e64239_app1.zip › S7 Number and percentage of RS score responders per time-point and number of active modules.png]

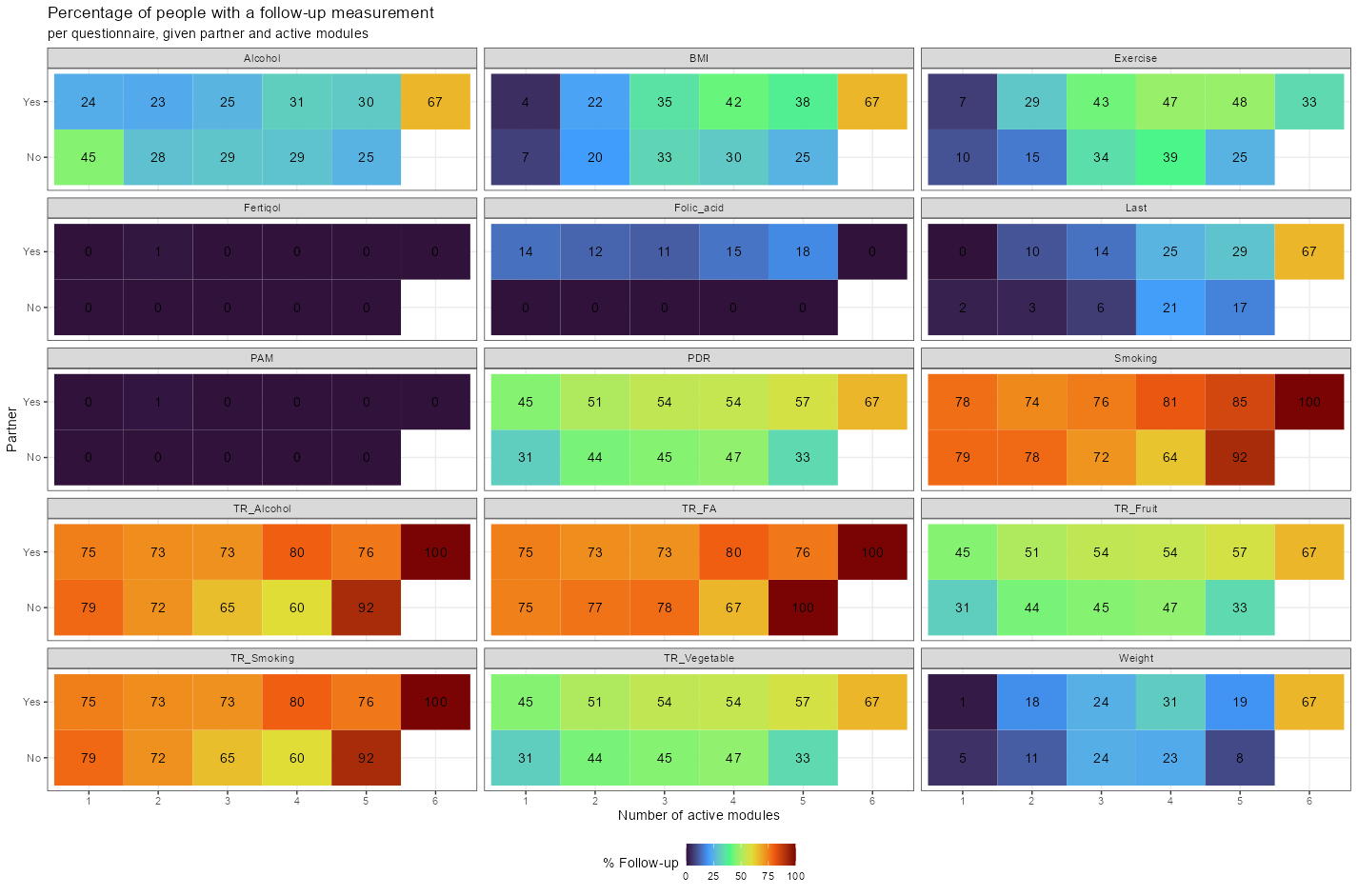

Supplement: Multimedia Appendix 1 [file formative_v9i1e64239_app1.zip › S8 Percentage followup by partner.png]

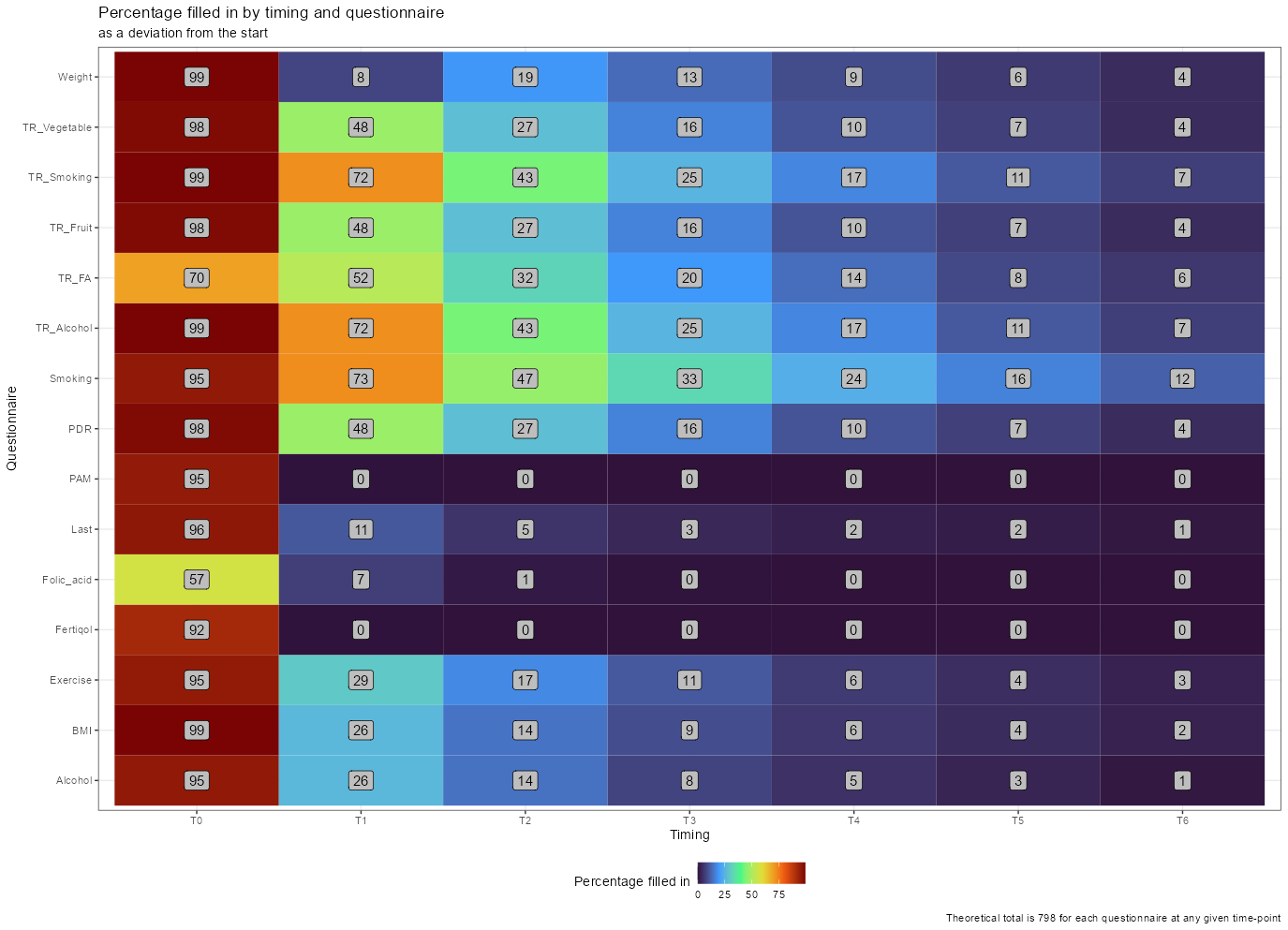

Supplement: Multimedia Appendix 1 [file formative_v9i1e64239_app1.zip › S9 Percentage filled in.png]

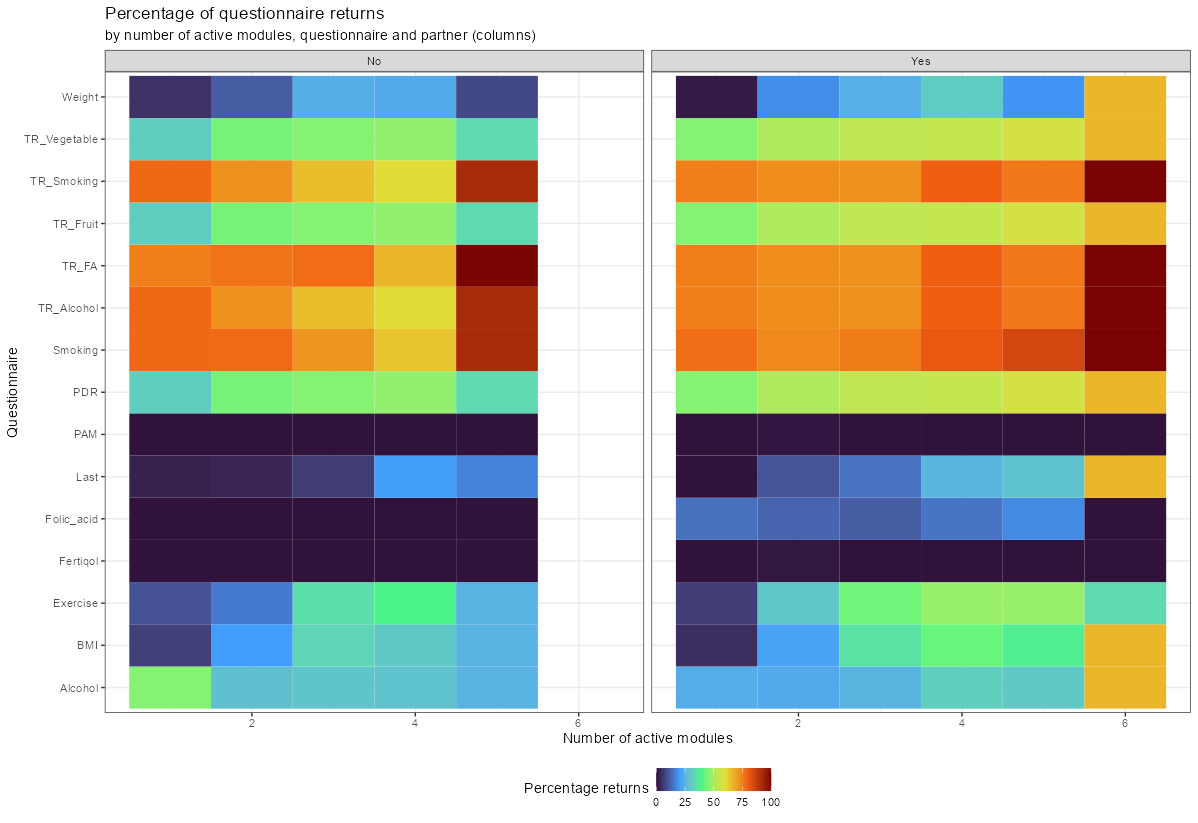

Supplement: Multimedia Appendix 1 [file formative_v9i1e64239_app1.zip › S10 Percentage of questionnaire returns.png]

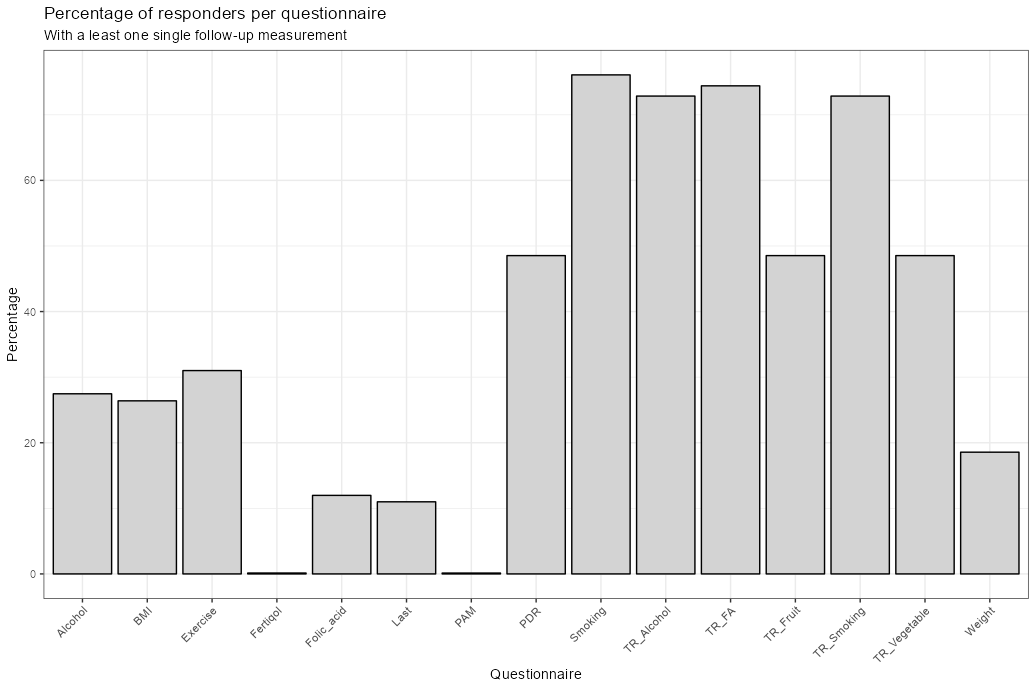

Supplement: Multimedia Appendix 1 [file formative_v9i1e64239_app1.zip › S11 Percentage of responders per questionnaire.png]

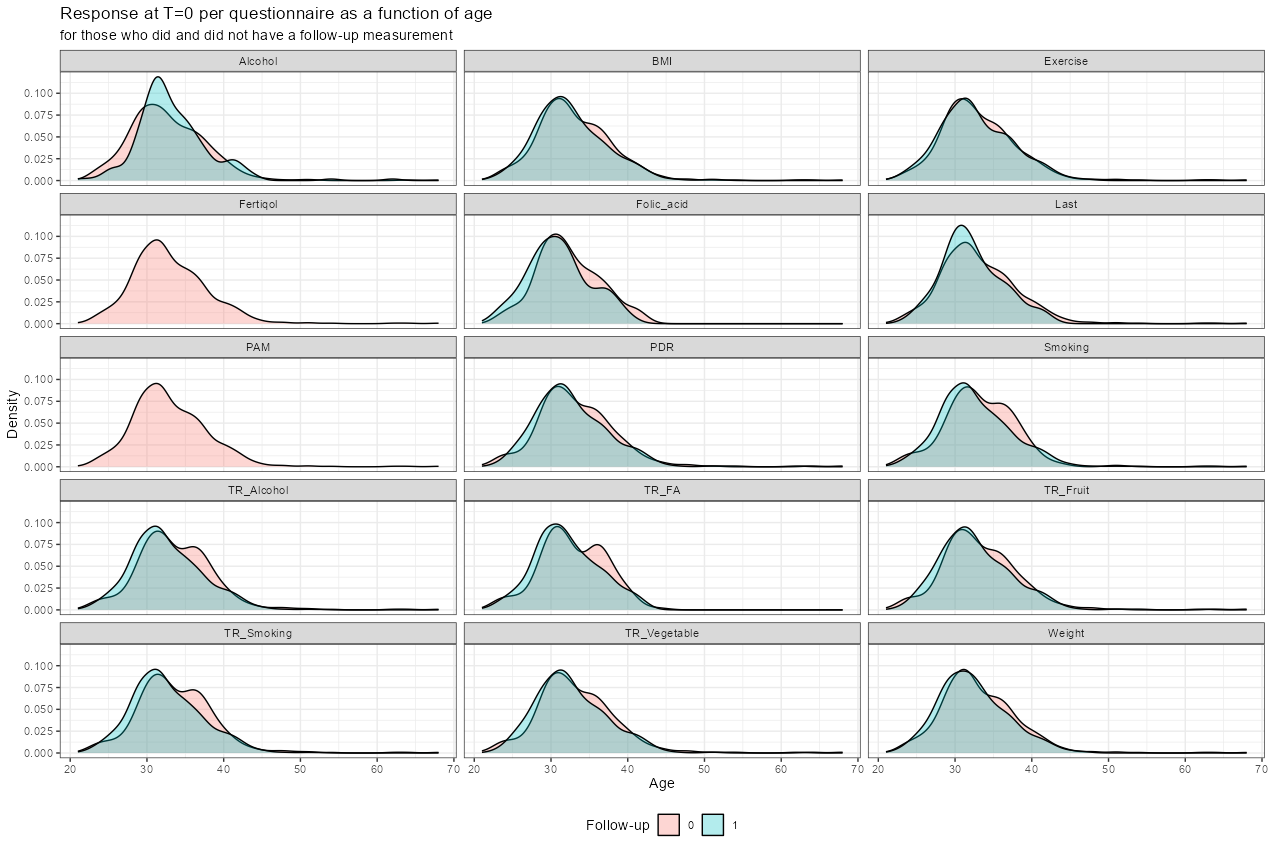

Supplement: Multimedia Appendix 1 [file formative_v9i1e64239_app1.zip › S12 Response at T=0 per questionnaire as a function of age.png]

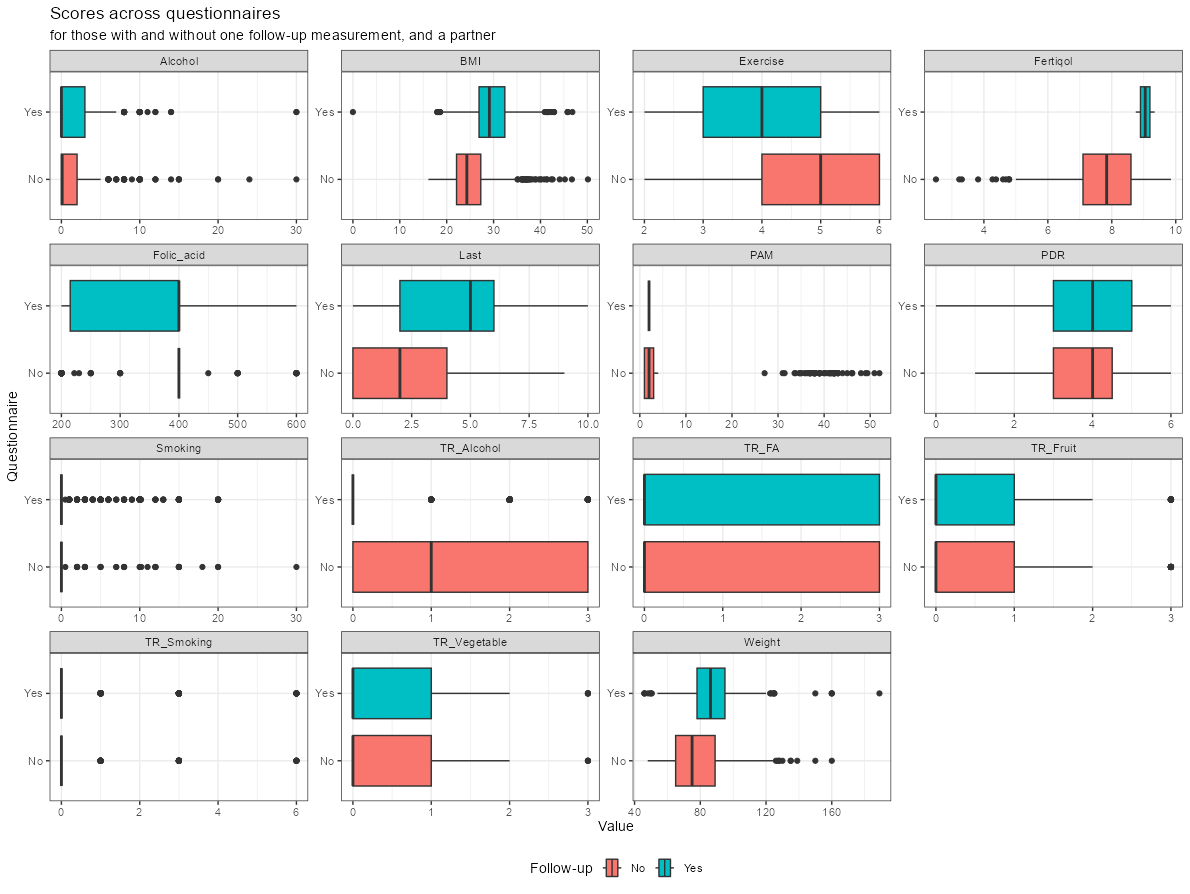

Supplement: Multimedia Appendix 1 [file formative_v9i1e64239_app1.zip › S13 Scores across questionnaires for those with and without one follow-up measurement.png]

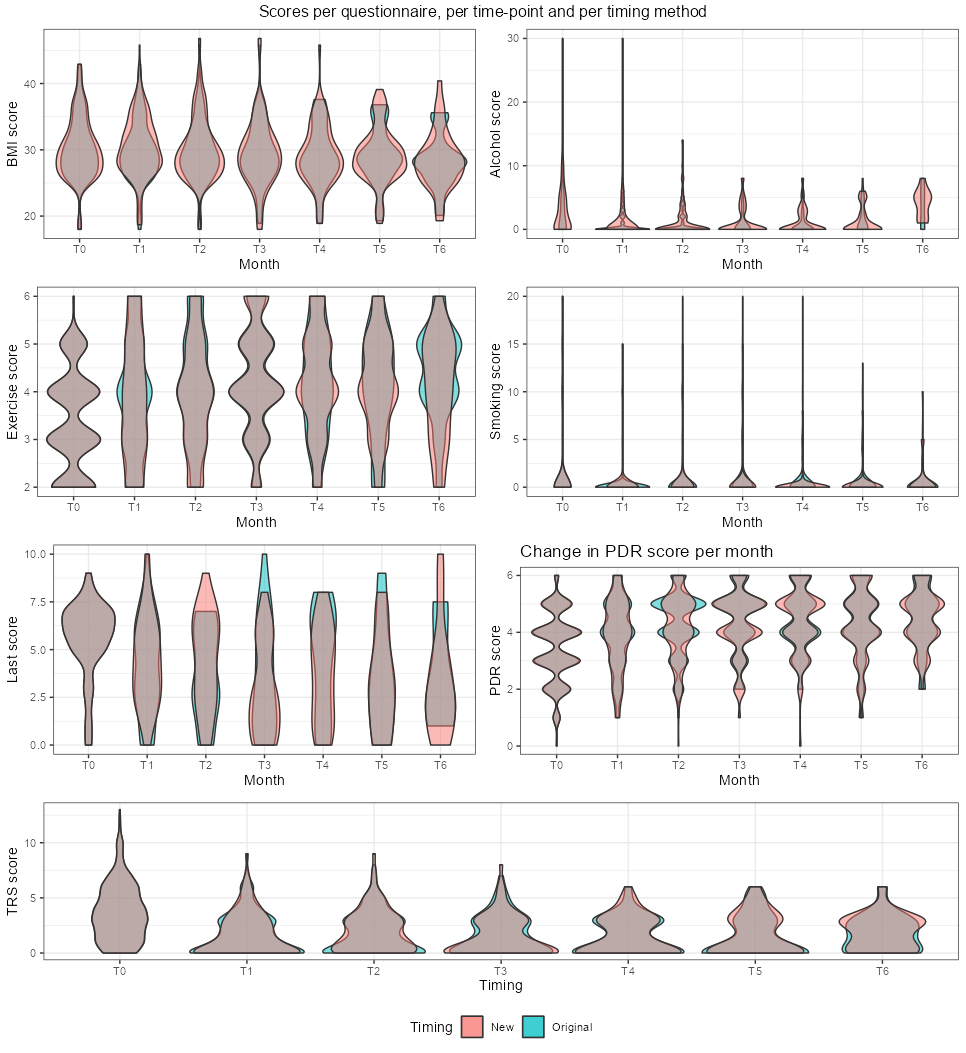

Supplement: Multimedia Appendix 1 [file formative_v9i1e64239_app1.zip › S14 Scores per questionnaire, per time-point and per timing method.png]

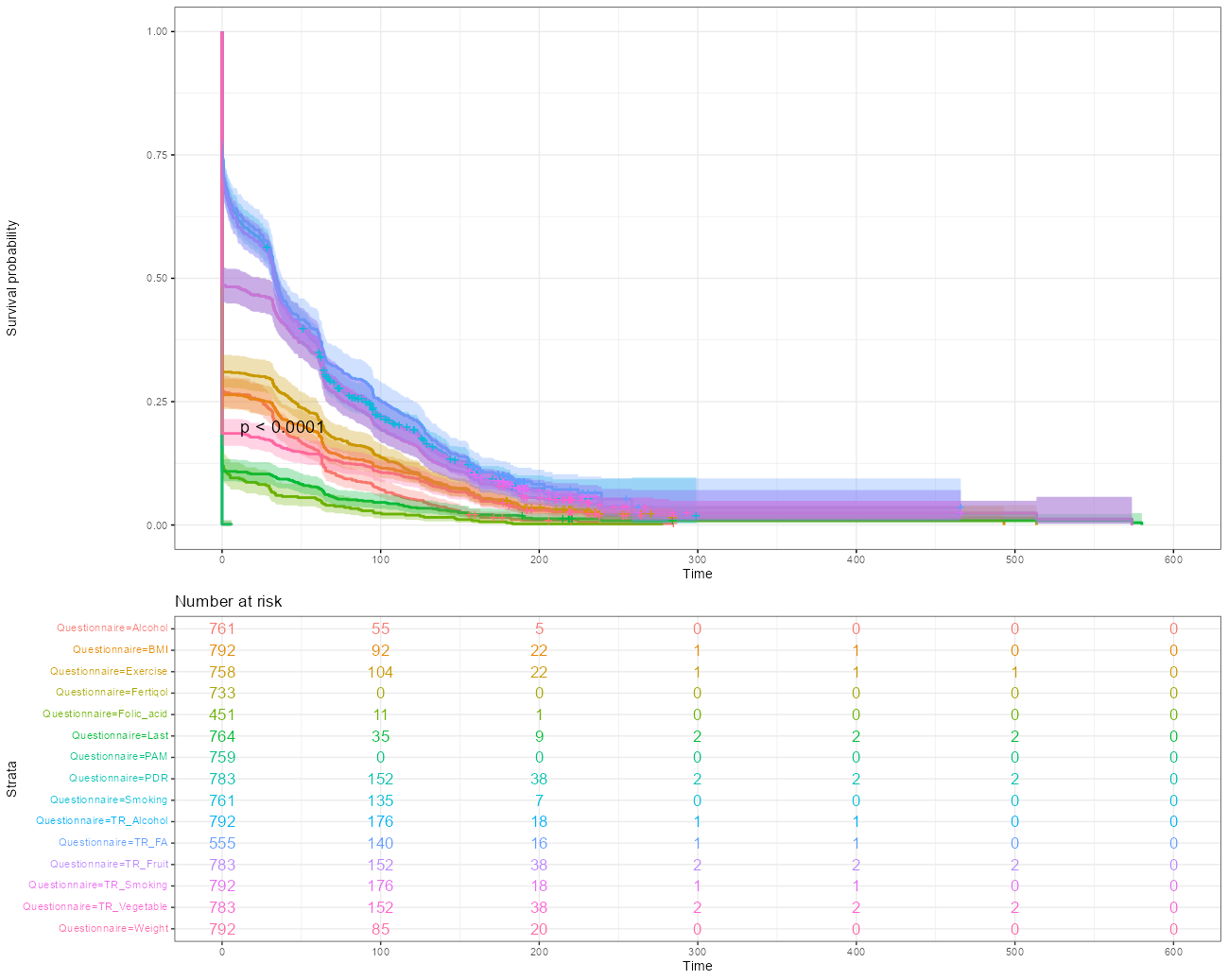

Supplement: Multimedia Appendix 1 [file formative_v9i1e64239_app1.zip › S15 Survival questionnaire response.png]

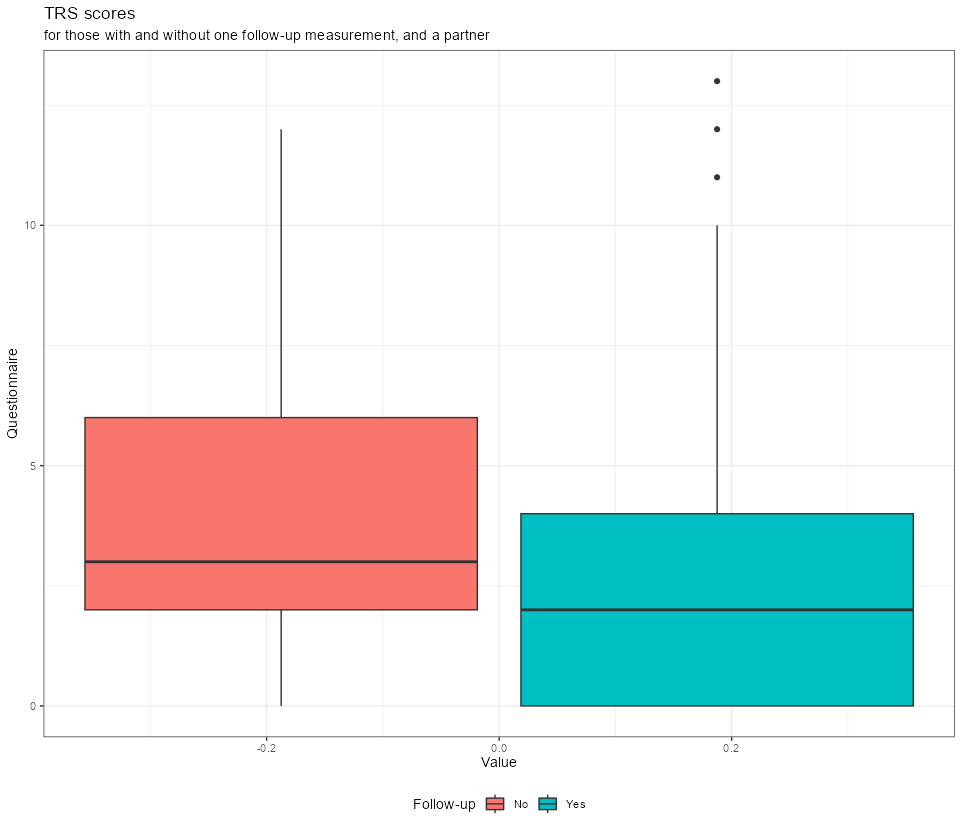

Supplement: Multimedia Appendix 1 [file formative_v9i1e64239_app1.zip › S16 TRS Score for those with and without one follow-up measurement.png]

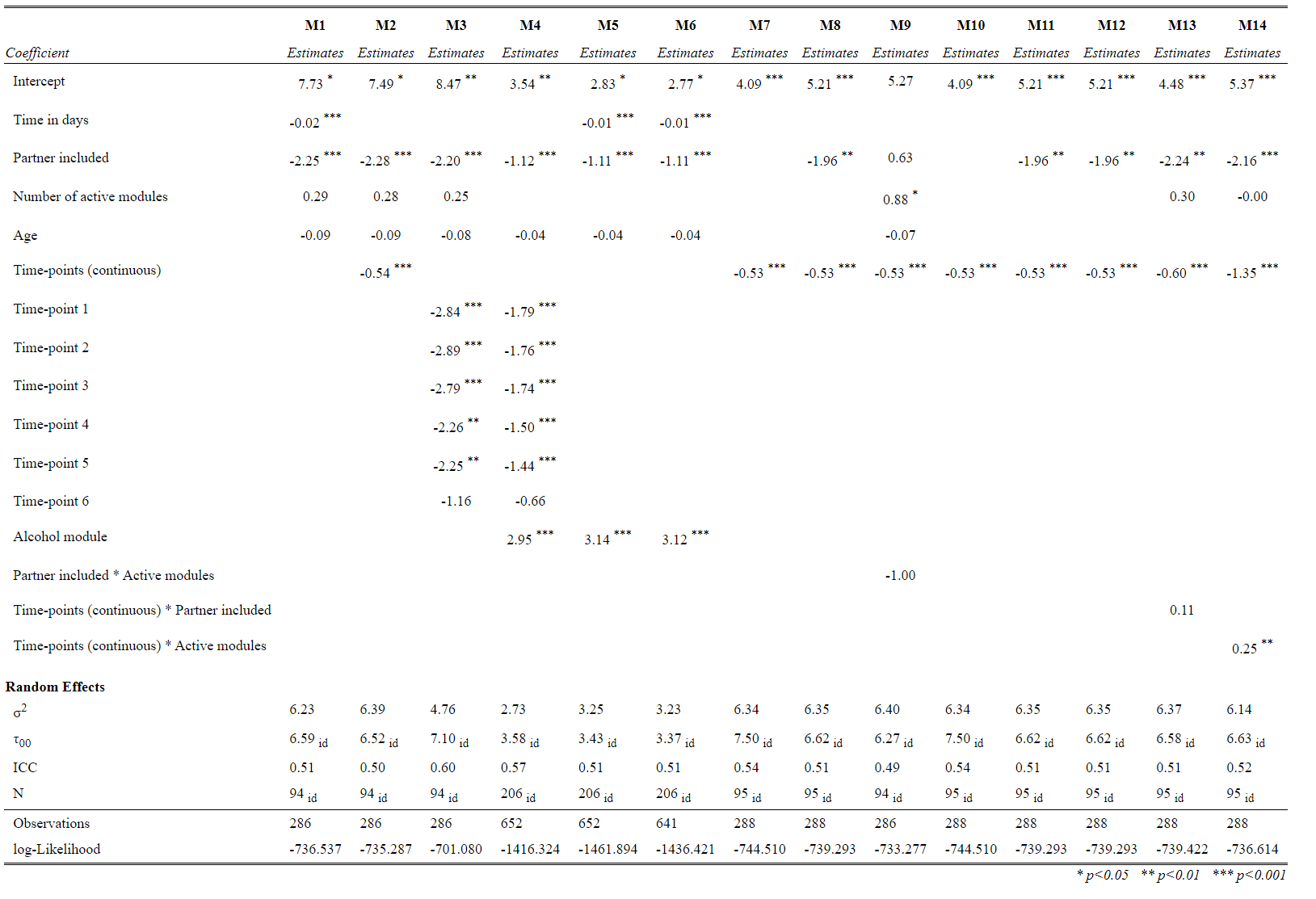

Supplement: Multimedia Appendix 1 [file formative_v9i1e64239_app1.zip › S17 Alcohol Model Selection.png]

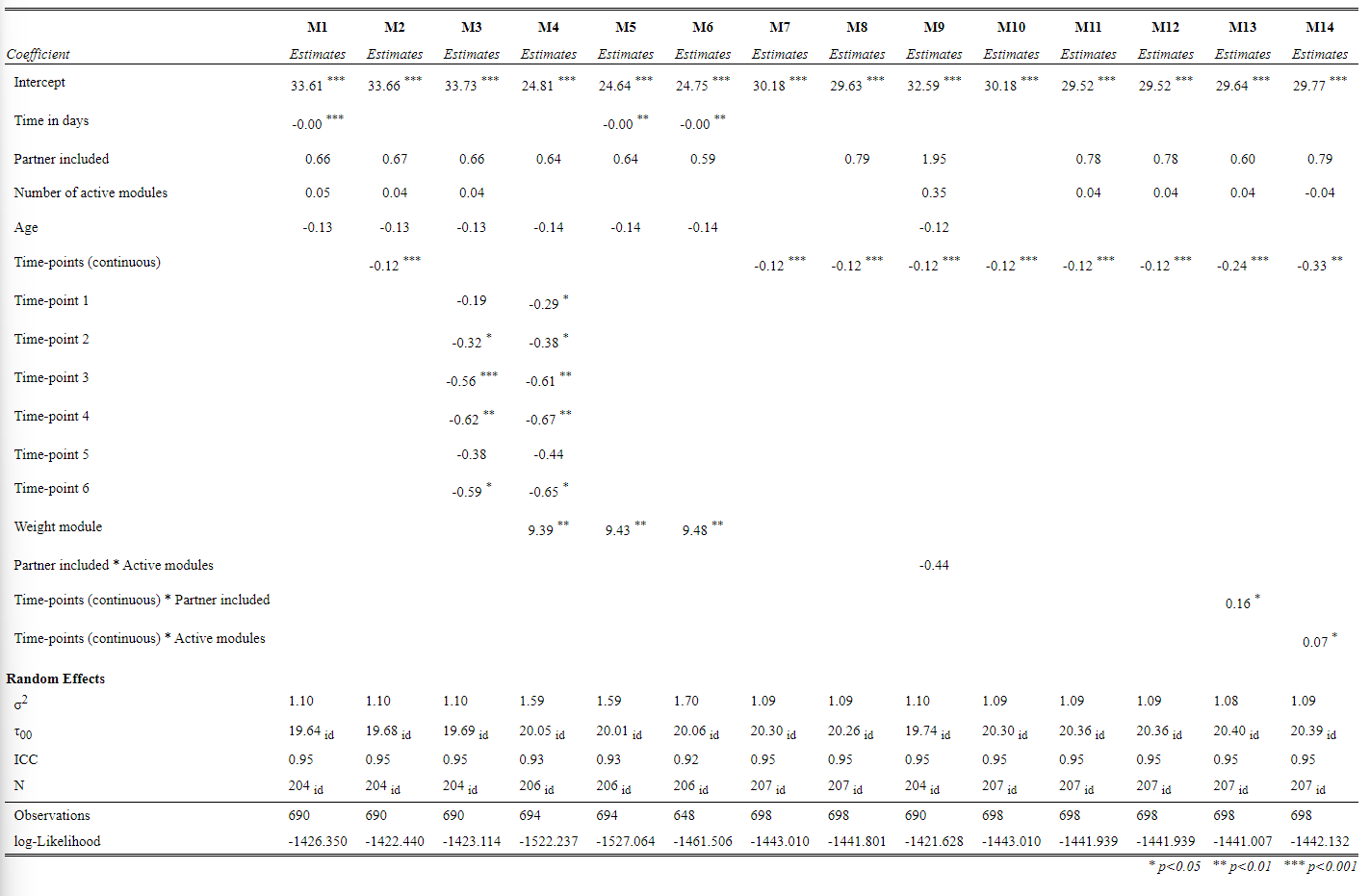

Supplement: Multimedia Appendix 1 [file formative_v9i1e64239_app1.zip › S18 BMI Model Selection.png]

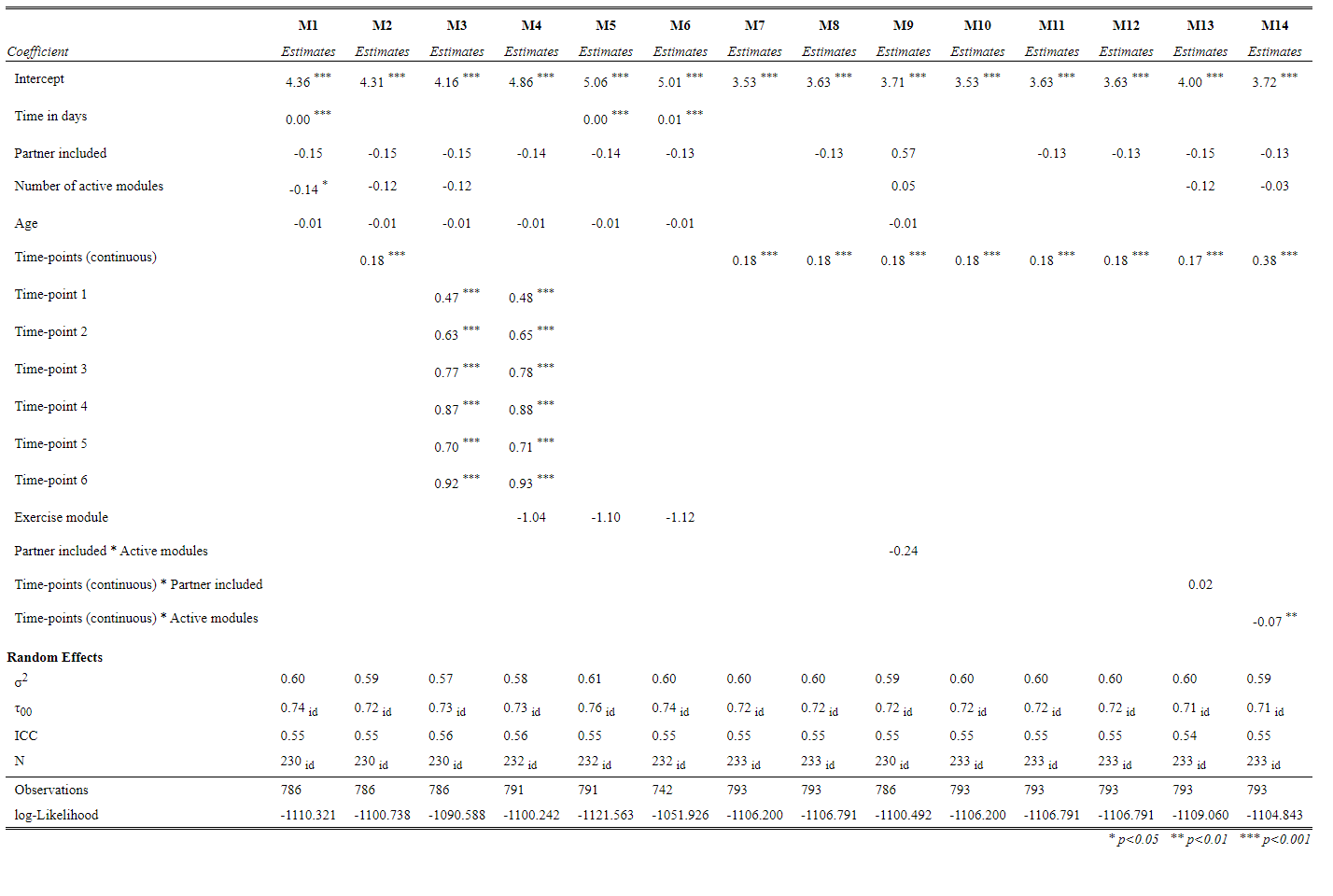

Supplement: Multimedia Appendix 1 [file formative_v9i1e64239_app1.zip › S19 Exercise Model Selection.png]

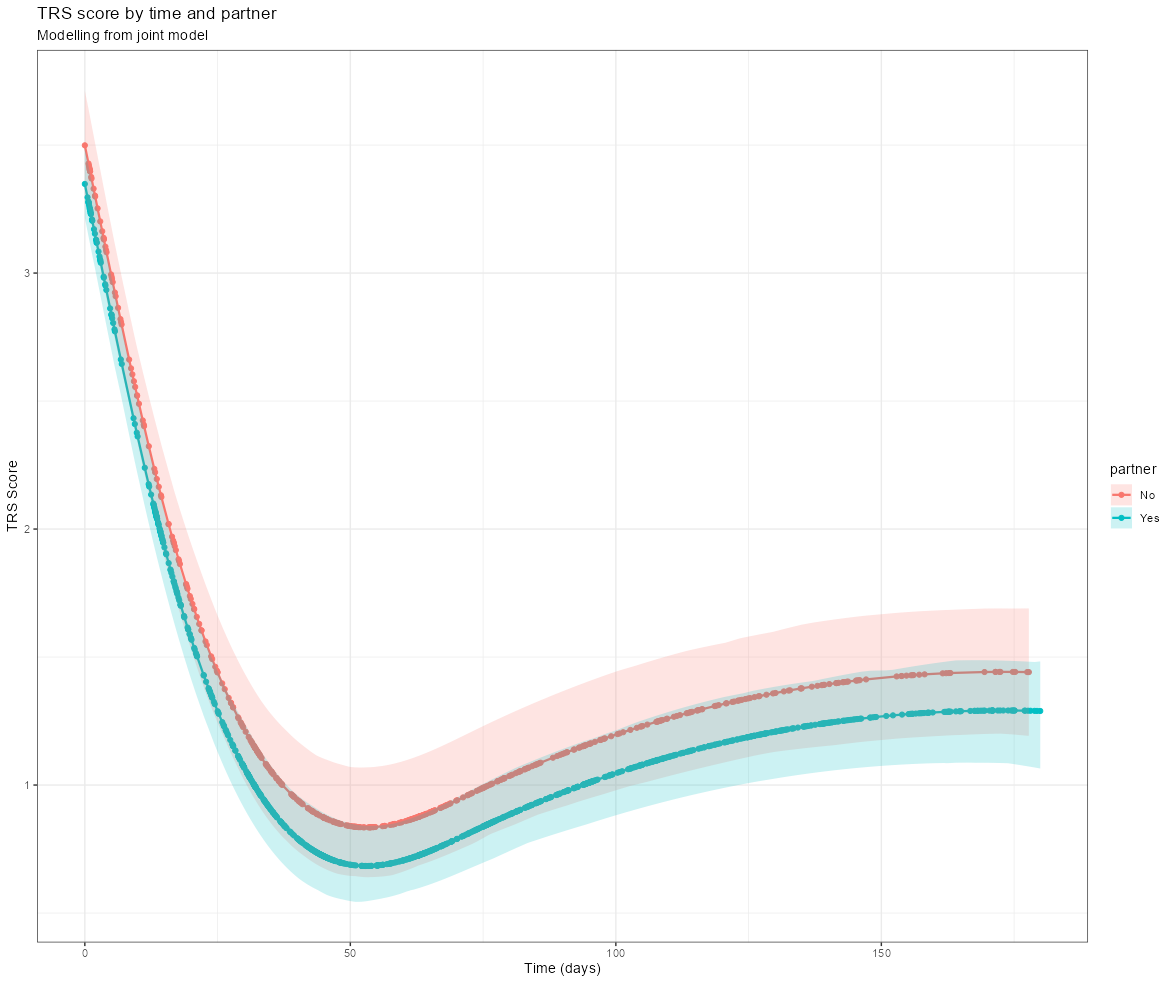

Supplement: Multimedia Appendix 1 [file formative_v9i1e64239_app1.zip › S19 Joint Model TRS.png]

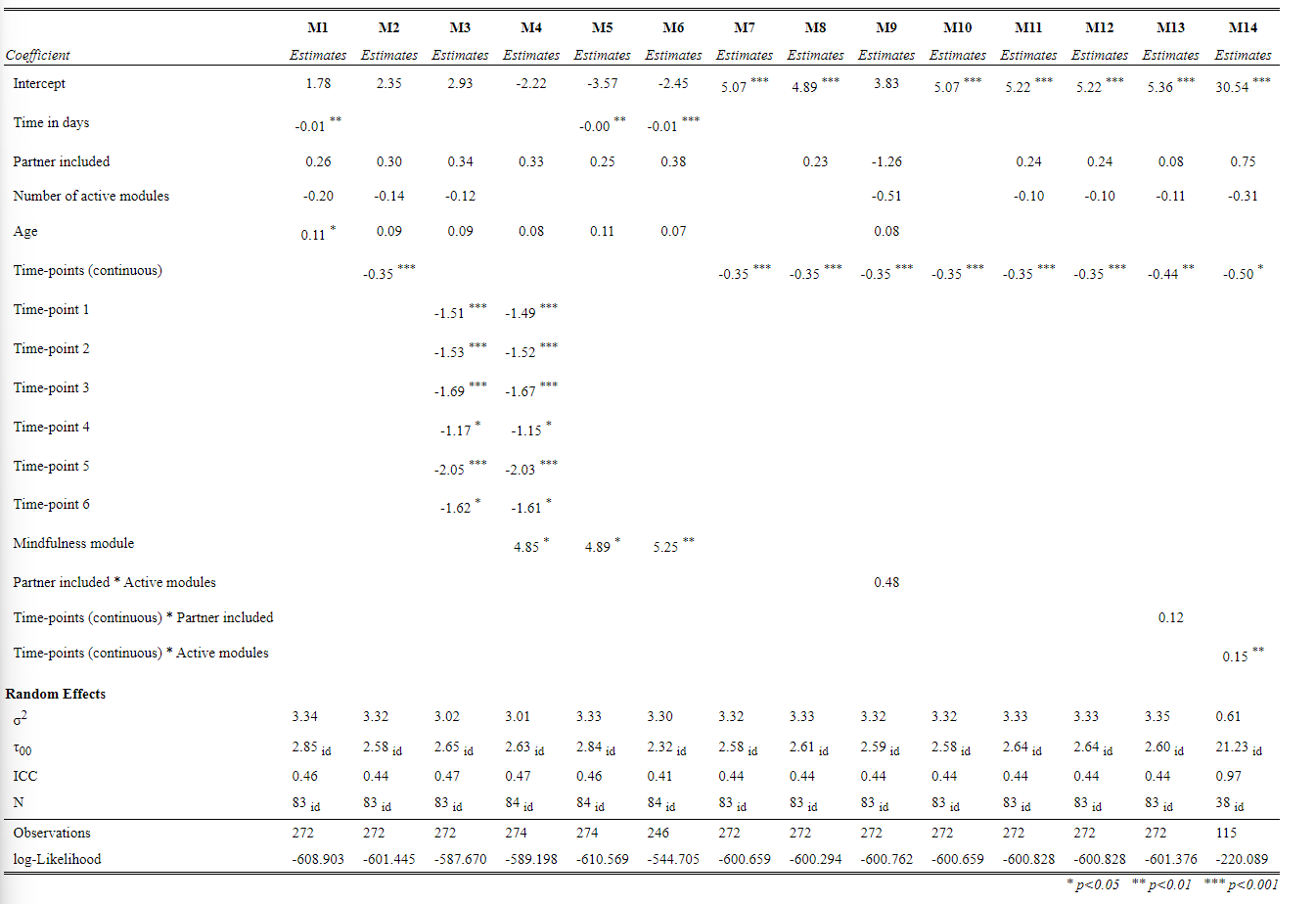

Supplement: Multimedia Appendix 1 [file formative_v9i1e64239_app1.zip › S20 LAST Model Selection.png]

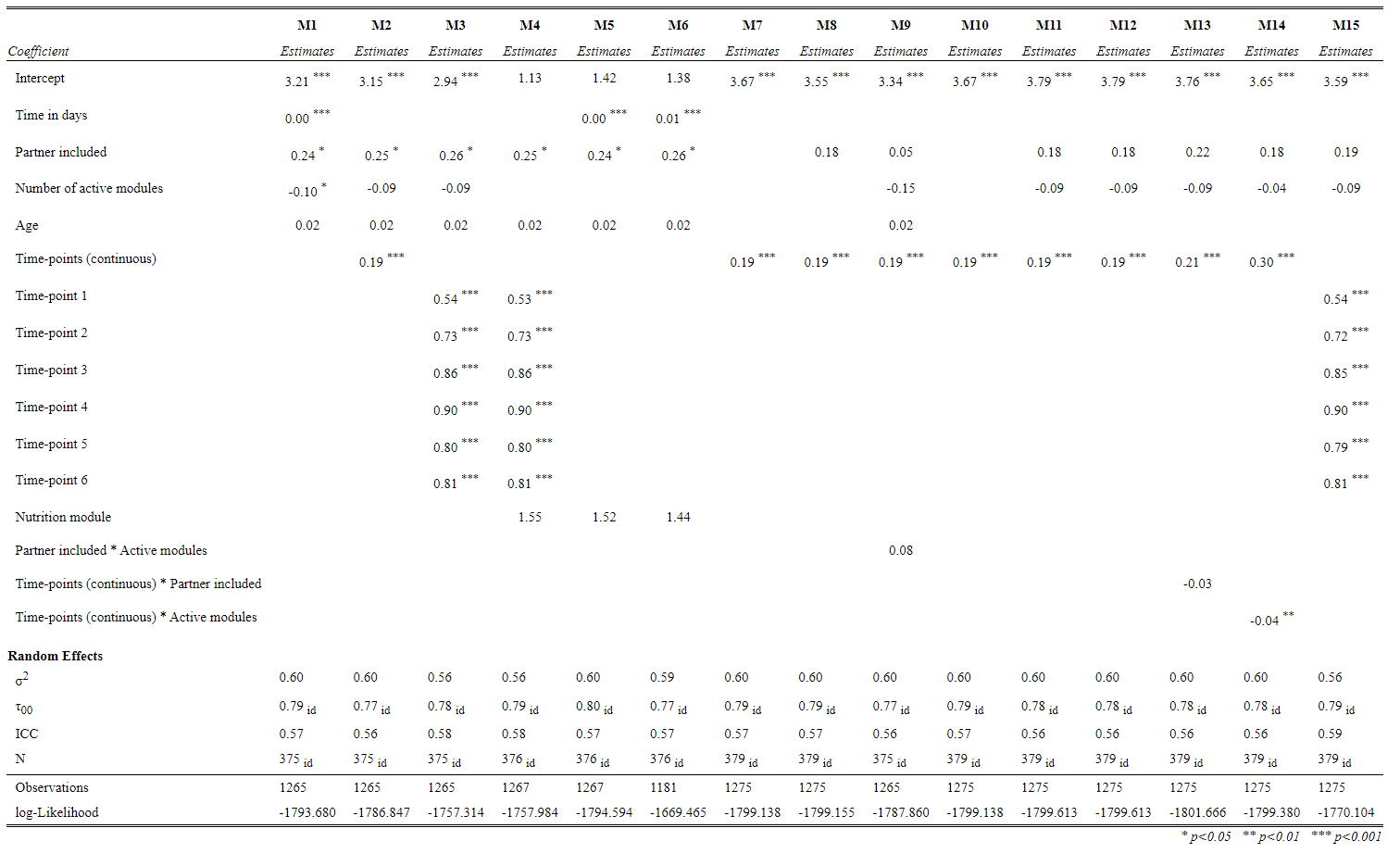

Supplement: Multimedia Appendix 1 [file formative_v9i1e64239_app1.zip › S21 PDR Model Selection.png]

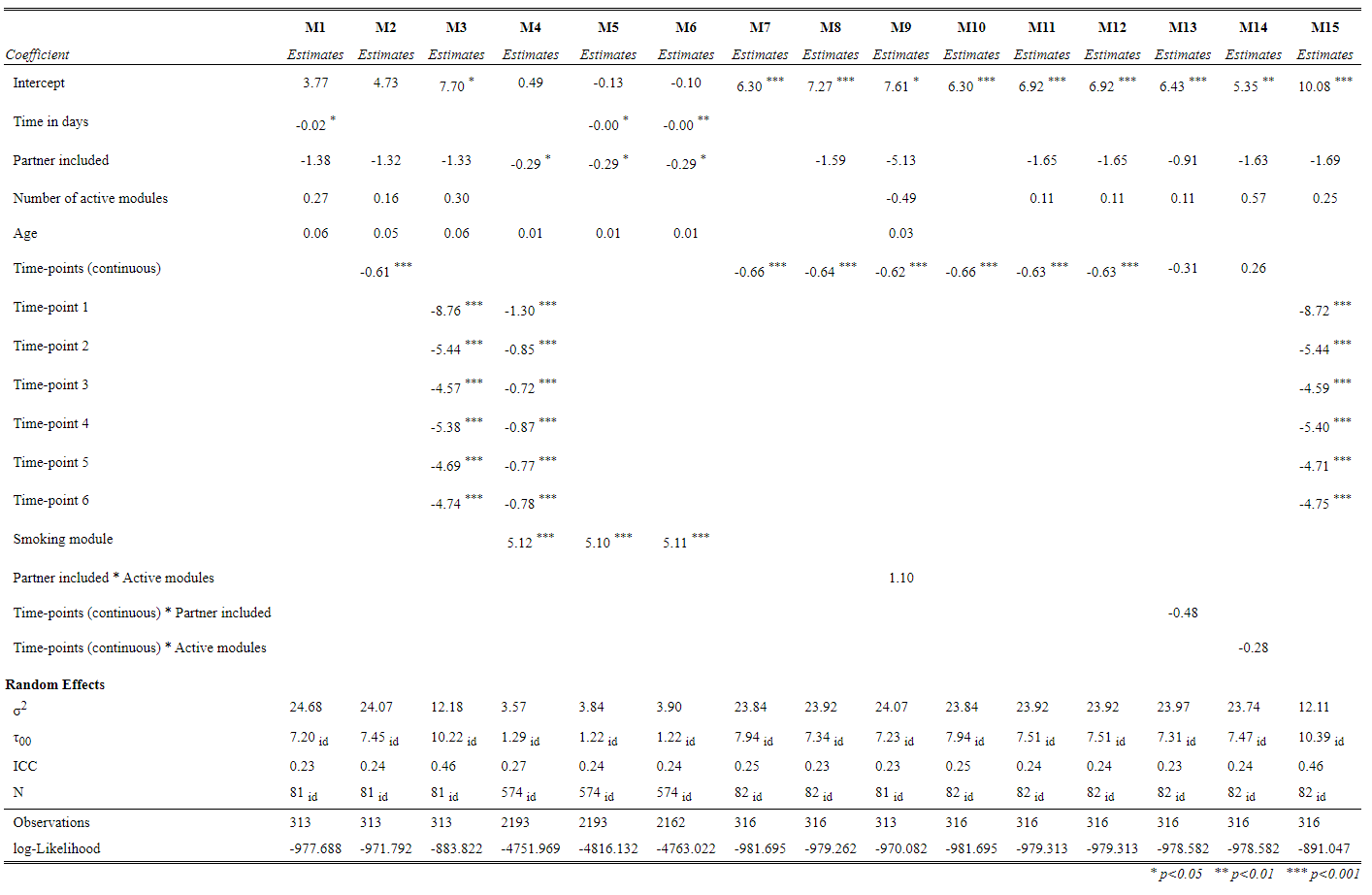

Supplement: Multimedia Appendix 1 [file formative_v9i1e64239_app1.zip › S22 Smoking Model Selection.png]

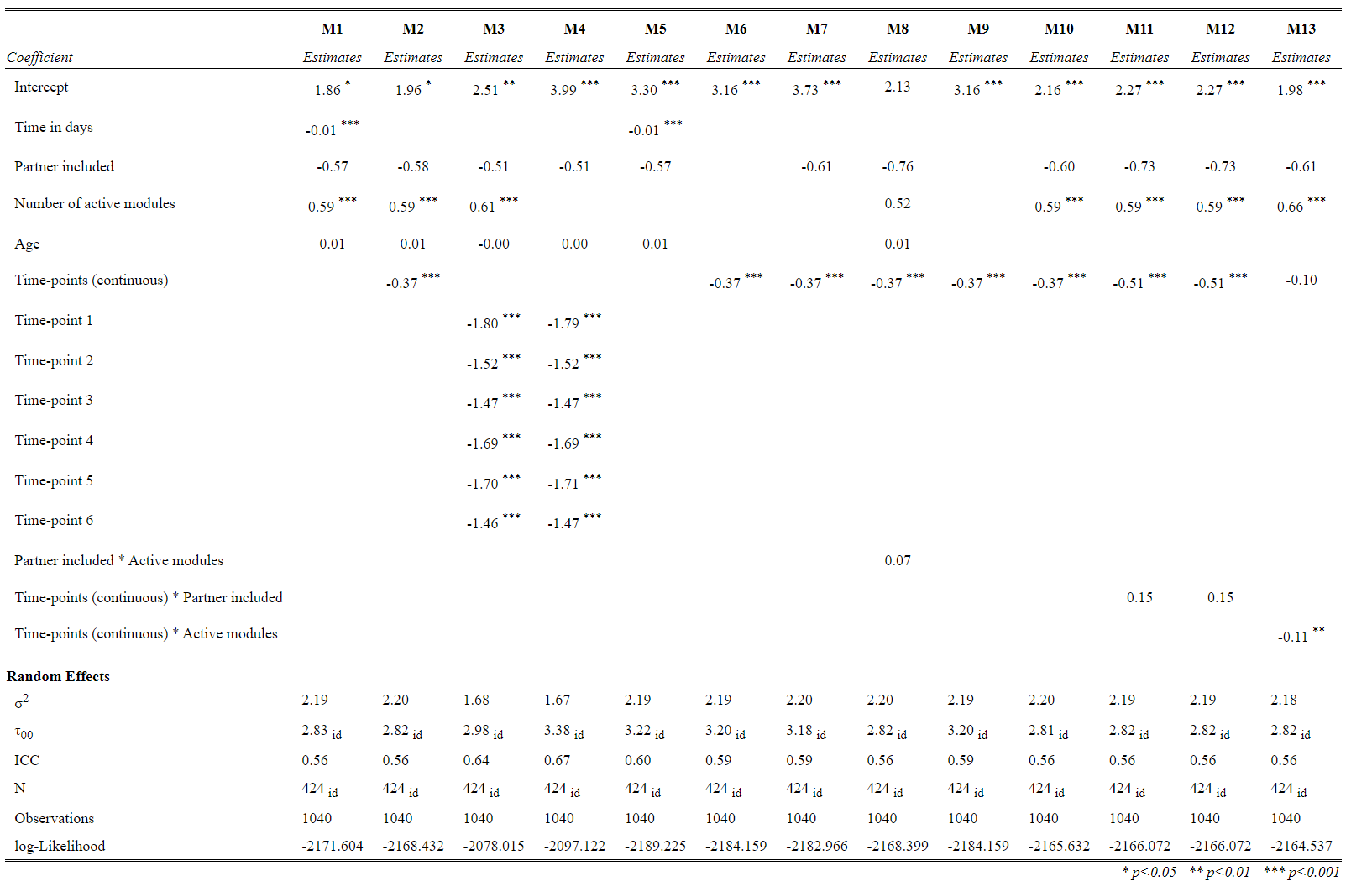

Supplement: Multimedia Appendix 1 [file formative_v9i1e64239_app1.zip › S23 TRS Model Selection.png]

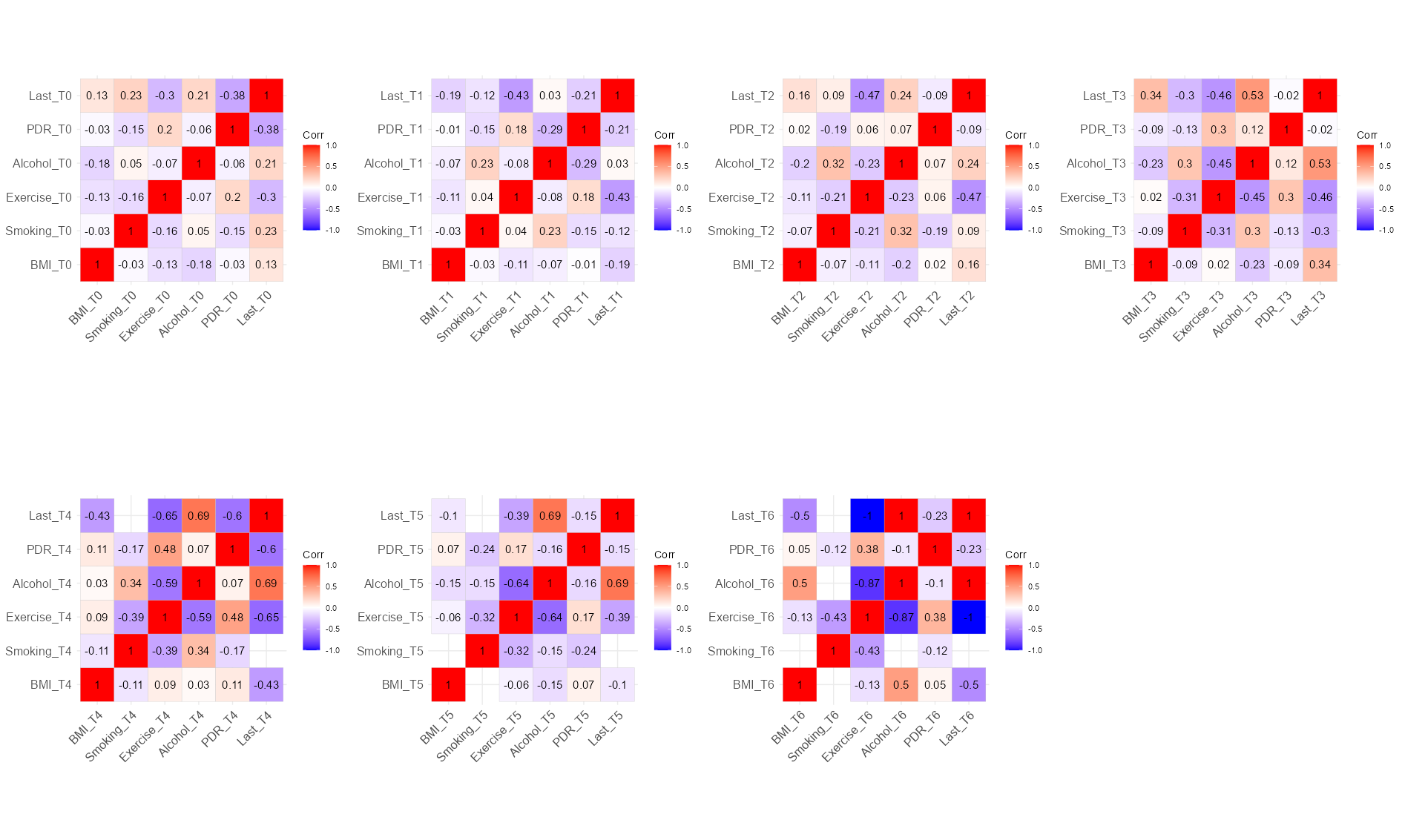

Supplement: Multimedia Appendix 1 [file formative_v9i1e64239_app1.zip › S24 Secondary Outcomes Correlation Matrix Across.png]

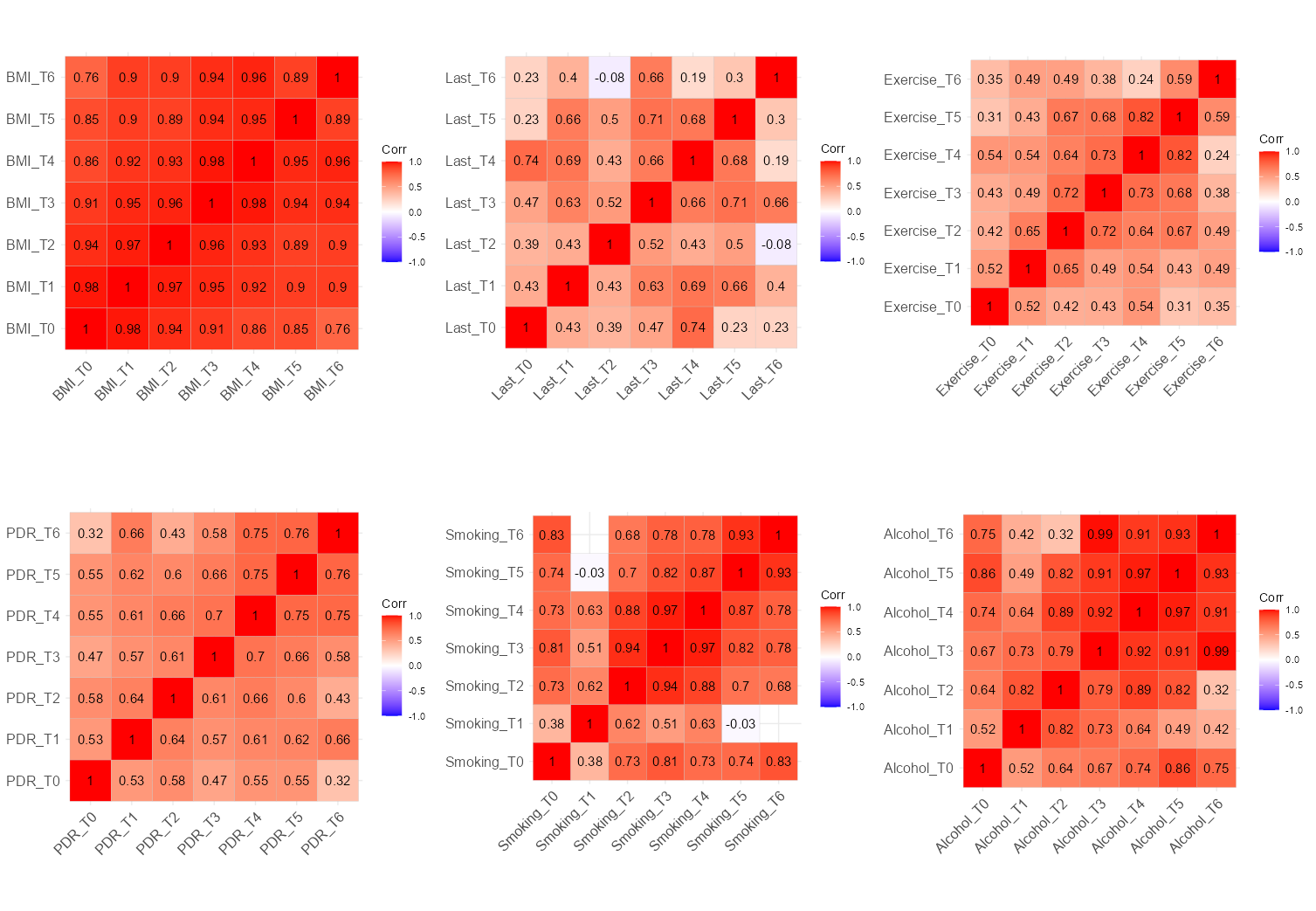

Supplement: Multimedia Appendix 1 [file formative_v9i1e64239_app1.zip › S25 Secondary Outcomes Correlation Matrix of Time.png]

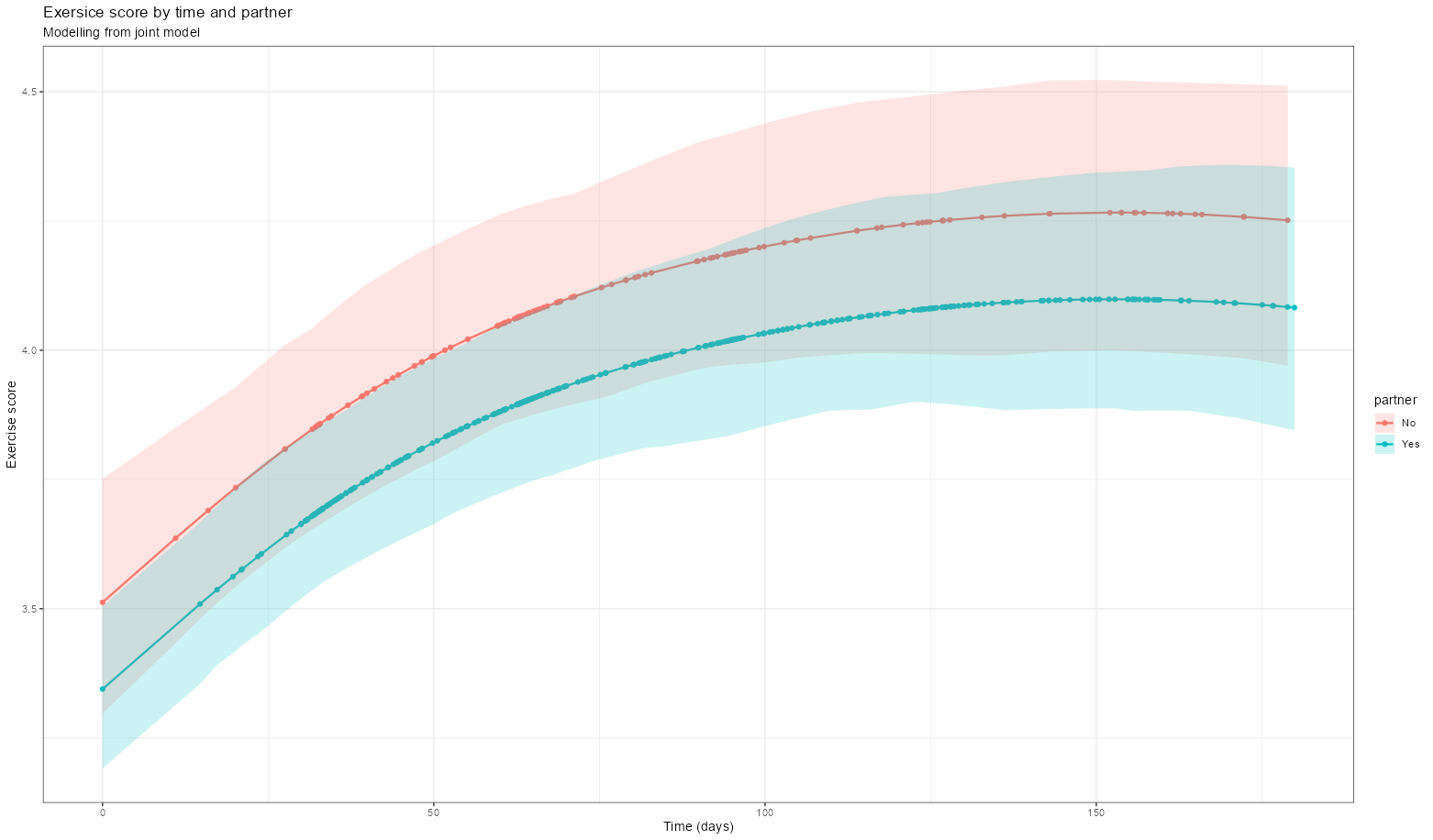

Supplement: Multimedia Appendix 1 [file formative_v9i1e64239_app1.zip › S26 JointModel Activity.png]

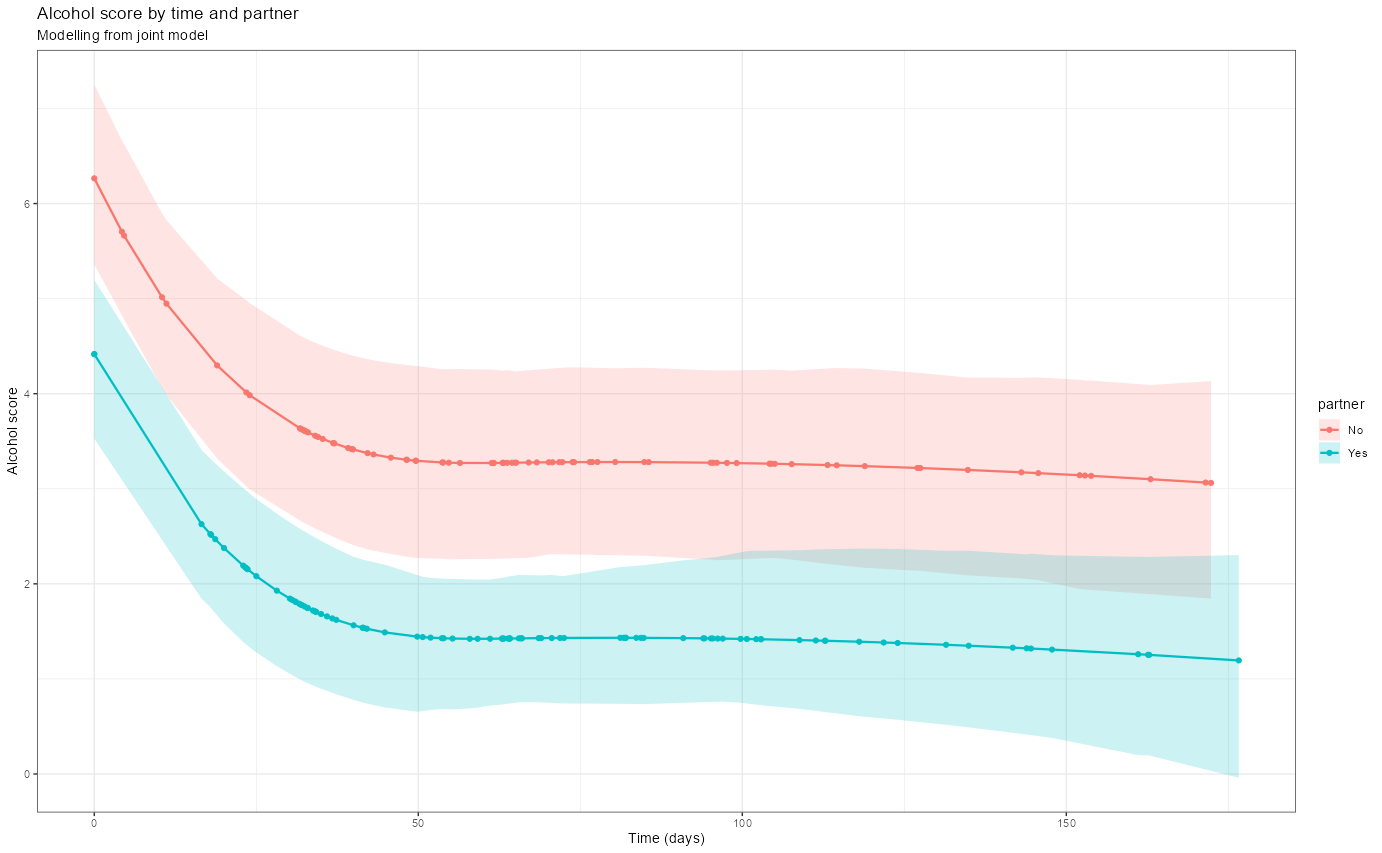

Supplement: Multimedia Appendix 1 [file formative_v9i1e64239_app1.zip › S27 Joint Model Alcohol.png]

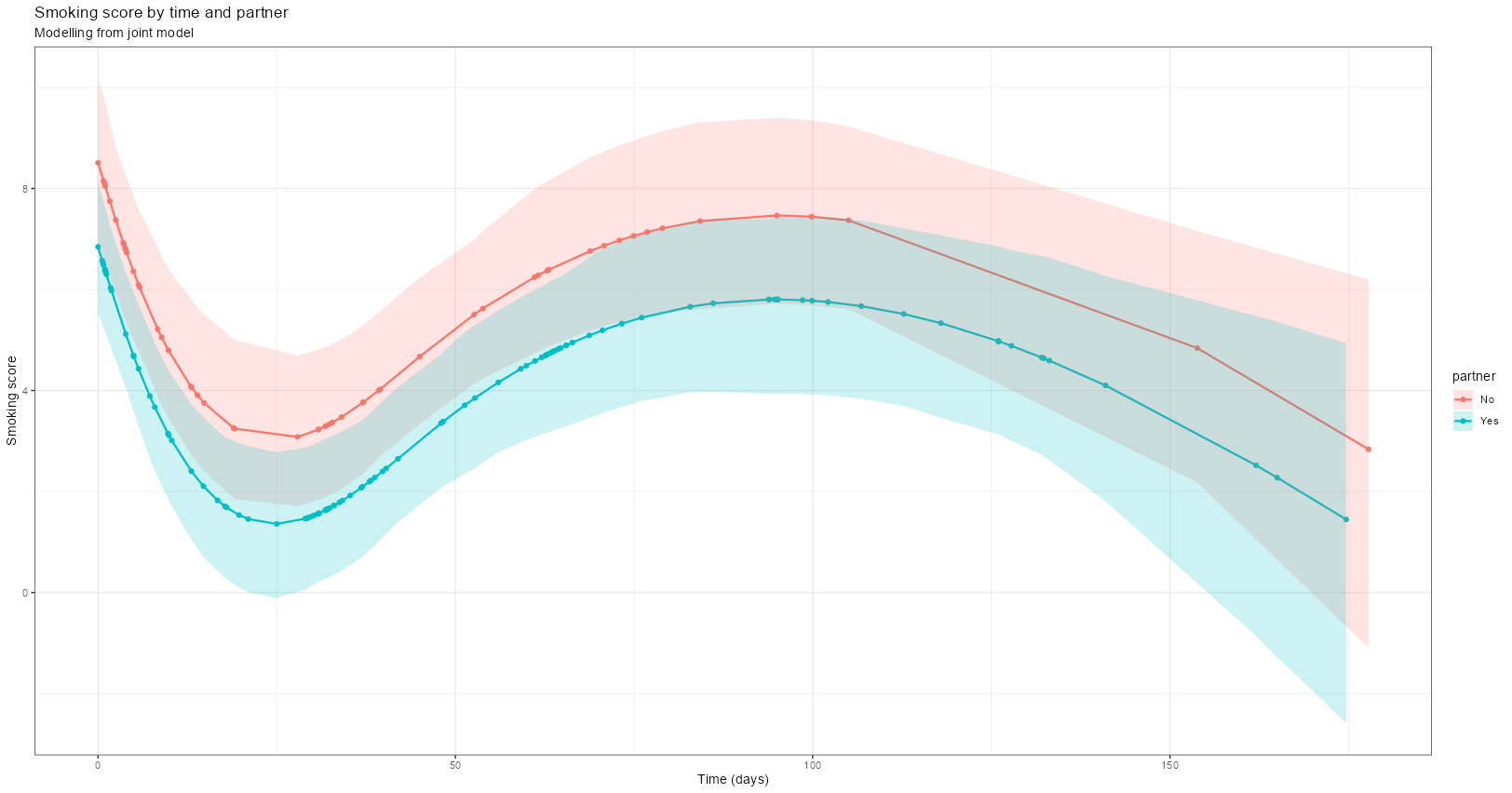

Supplement: Multimedia Appendix 1 [file formative_v9i1e64239_app1.zip › S28 Joint Model Smoking.png]

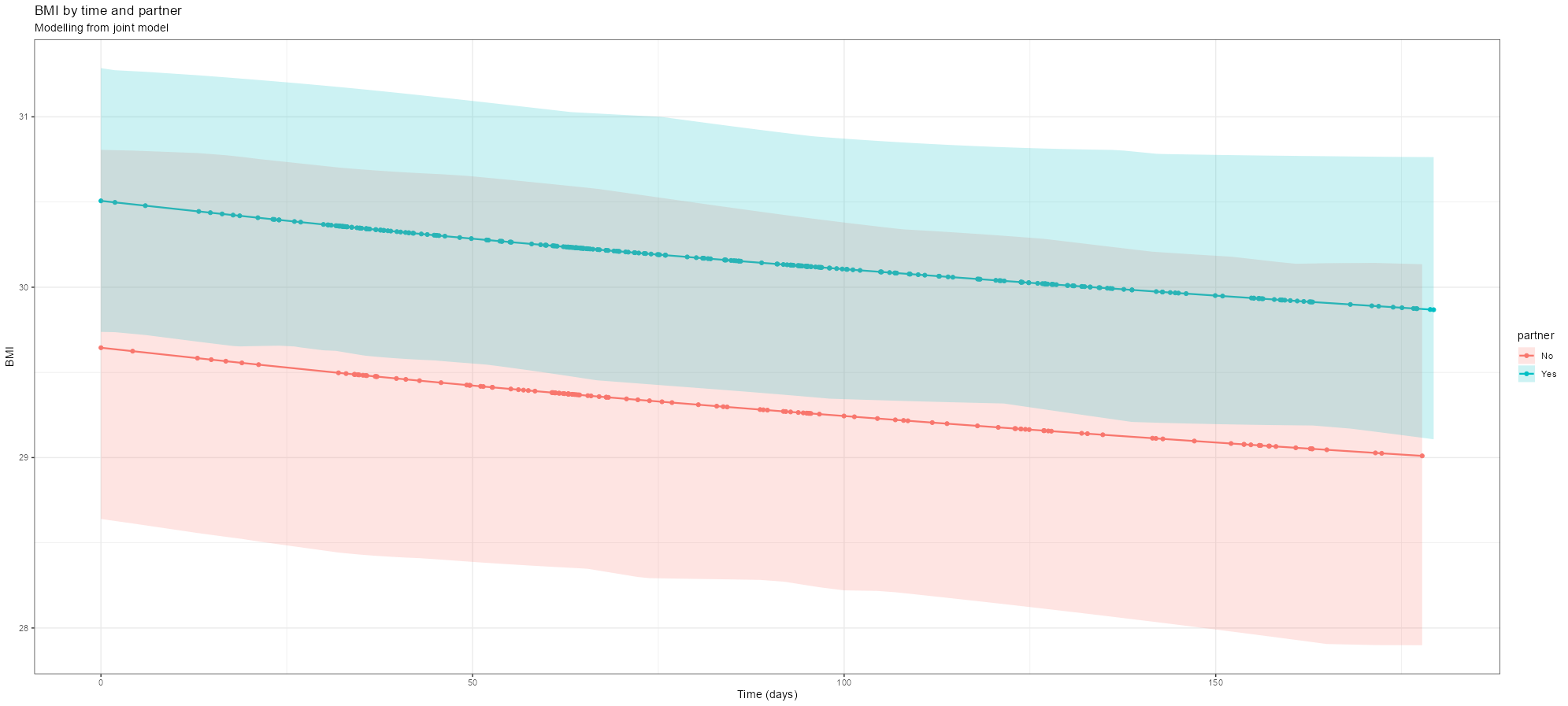

Supplement: Multimedia Appendix 1 [file formative_v9i1e64239_app1.zip › S29 BMI_JointModel.png]

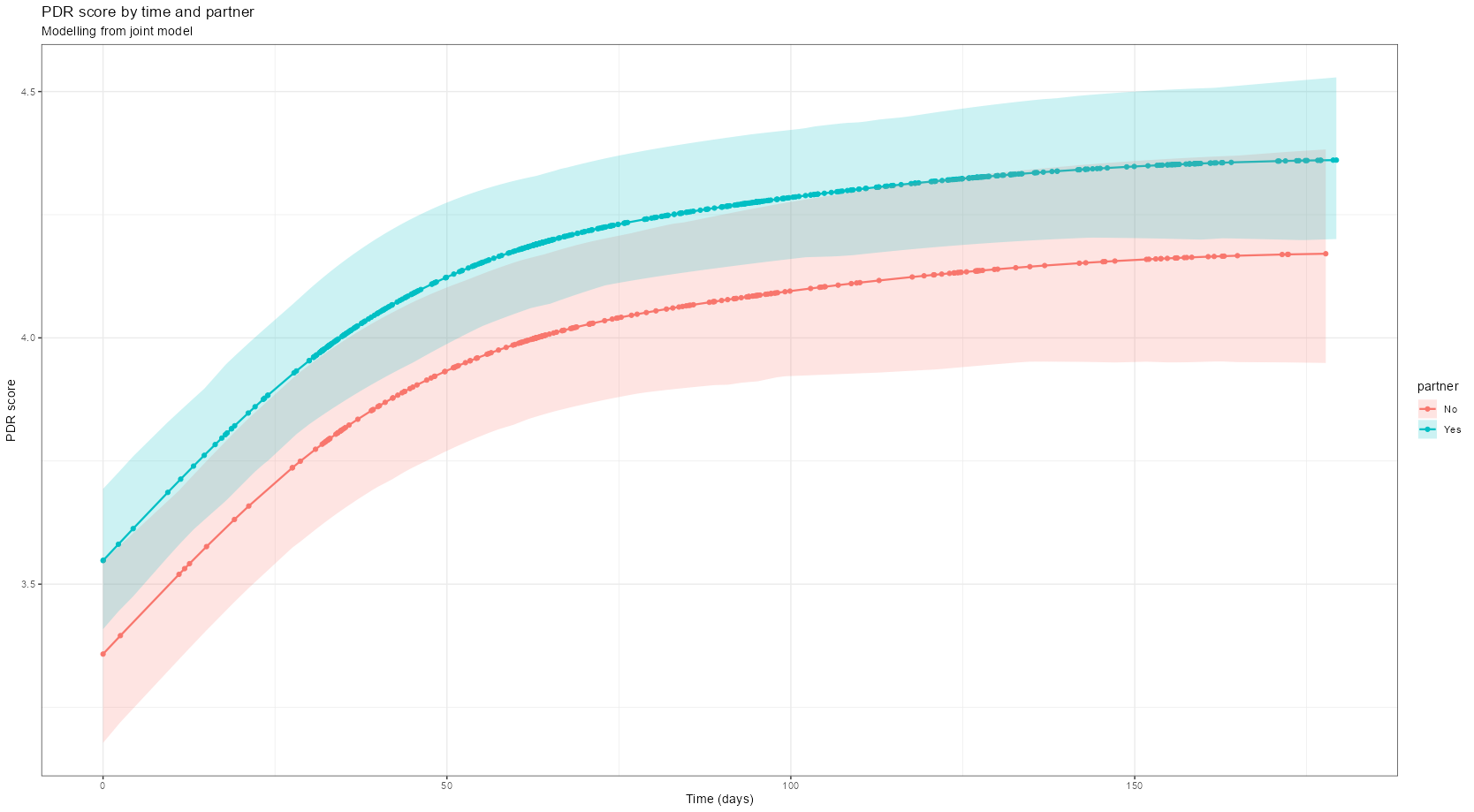

Supplement: Multimedia Appendix 1 [file formative_v9i1e64239_app1.zip › S30 Joint Model PDR.png]

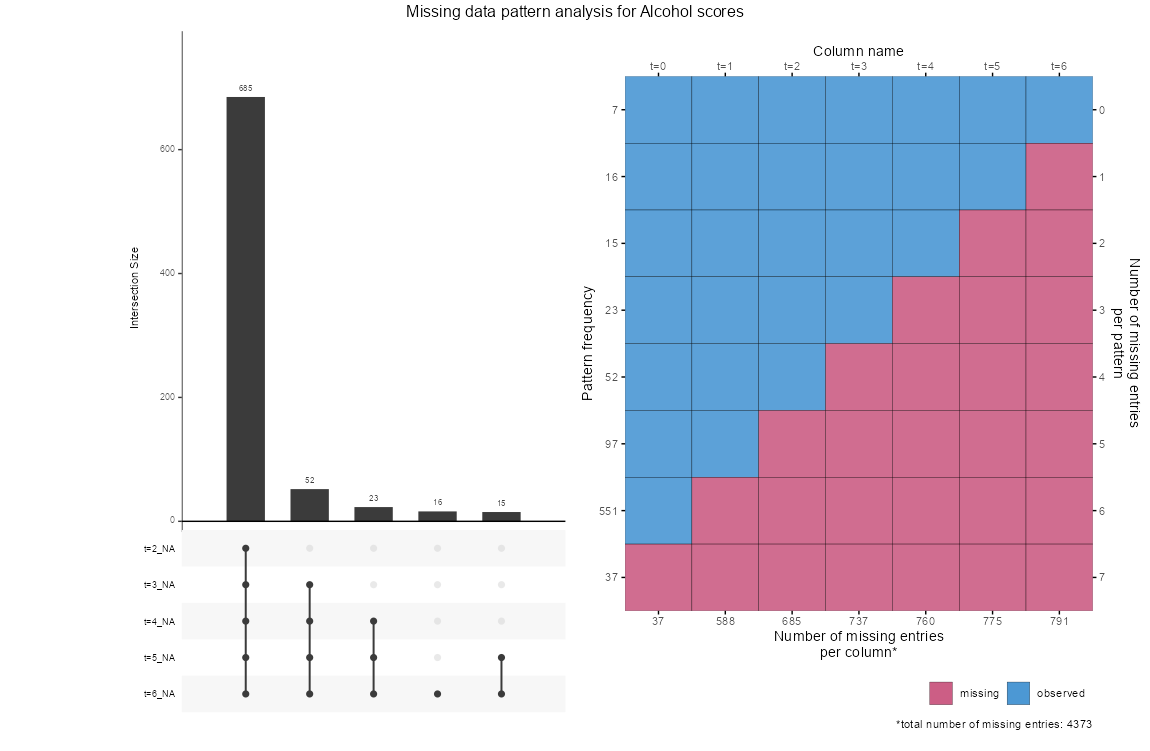

Supplement: Multimedia Appendix 1 [file formative_v9i1e64239_app1.zip › S31 Missing pattern analysis Alcohol.png]

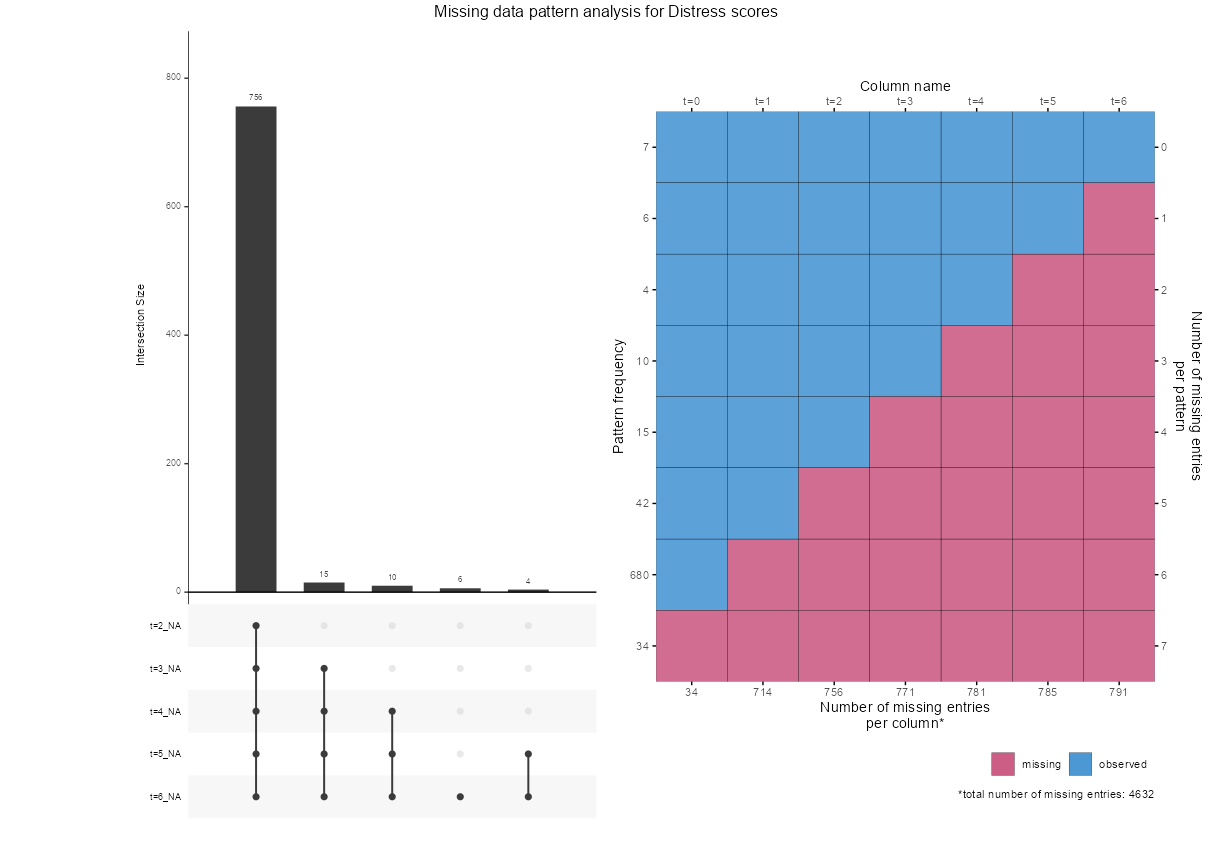

Supplement: Multimedia Appendix 1 [file formative_v9i1e64239_app1.zip › S32 Missing pattern analysis Distress.png]

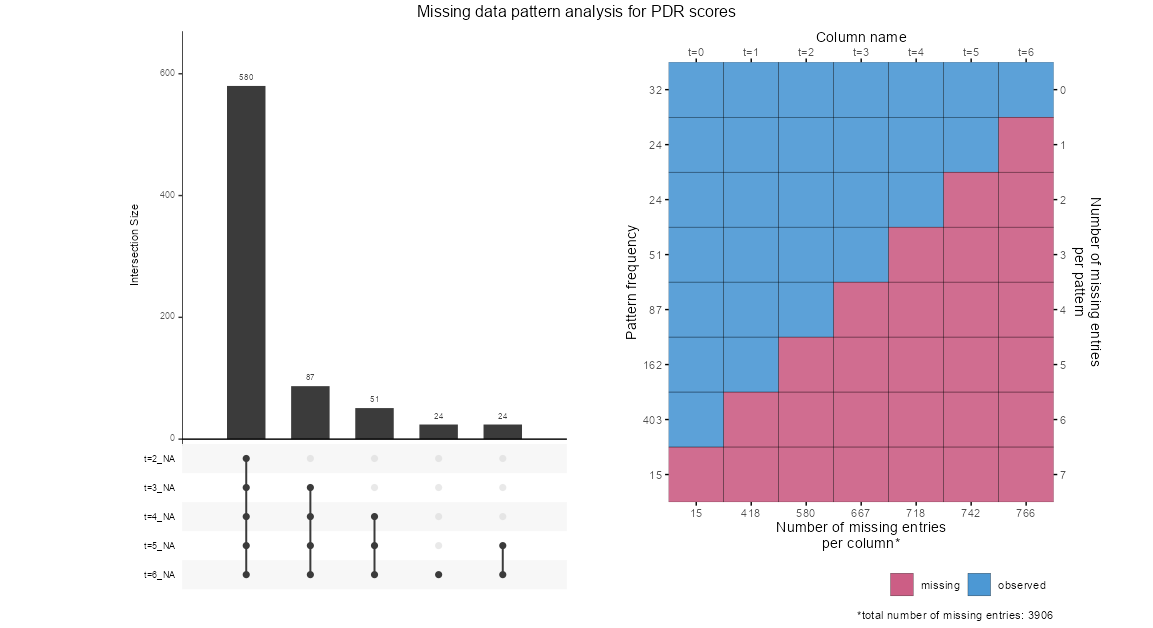

Supplement: Multimedia Appendix 1 [file formative_v9i1e64239_app1.zip › S33 Missing pattern analysis PDR.png]

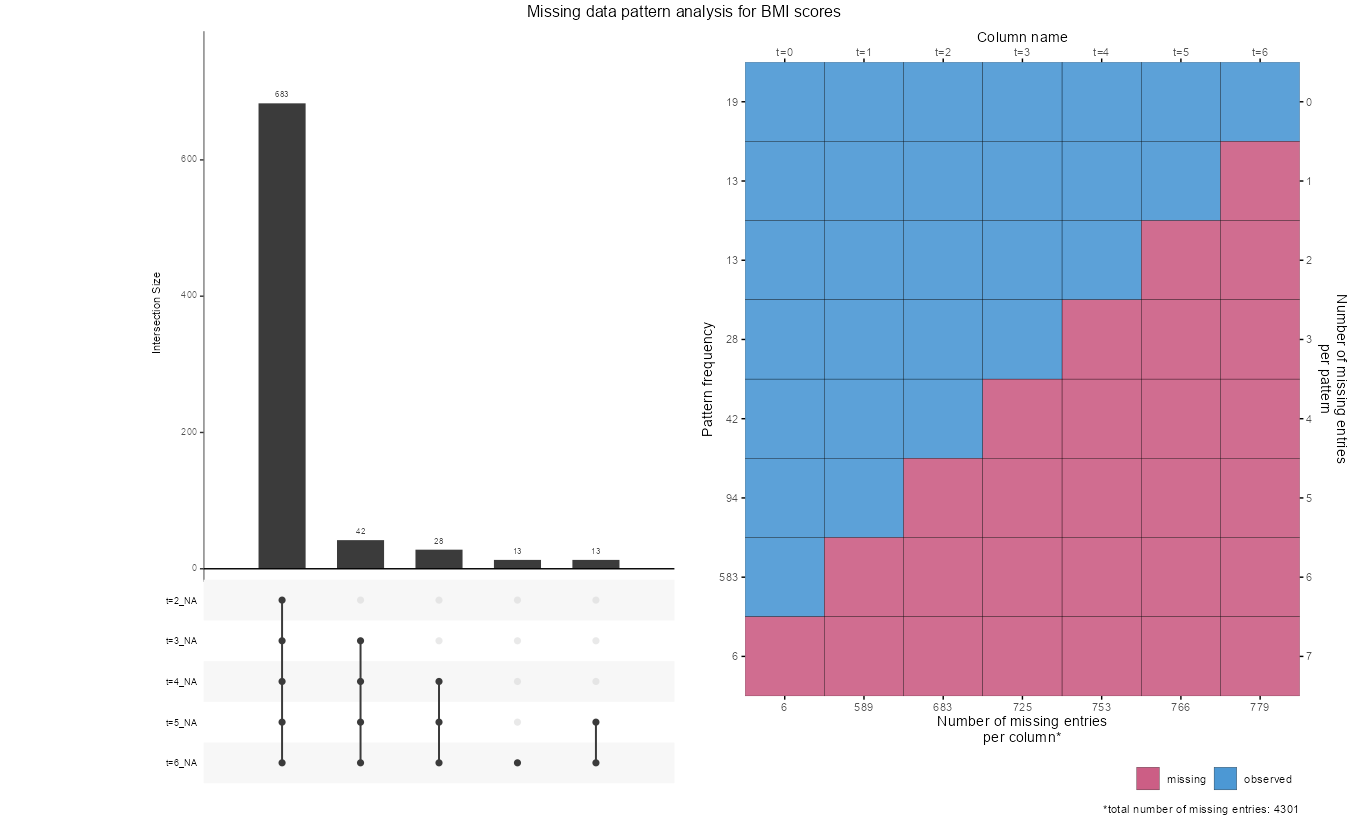

Supplement: Multimedia Appendix 1 [file formative_v9i1e64239_app1.zip › S34 Missing pattern analysis BMI.png]

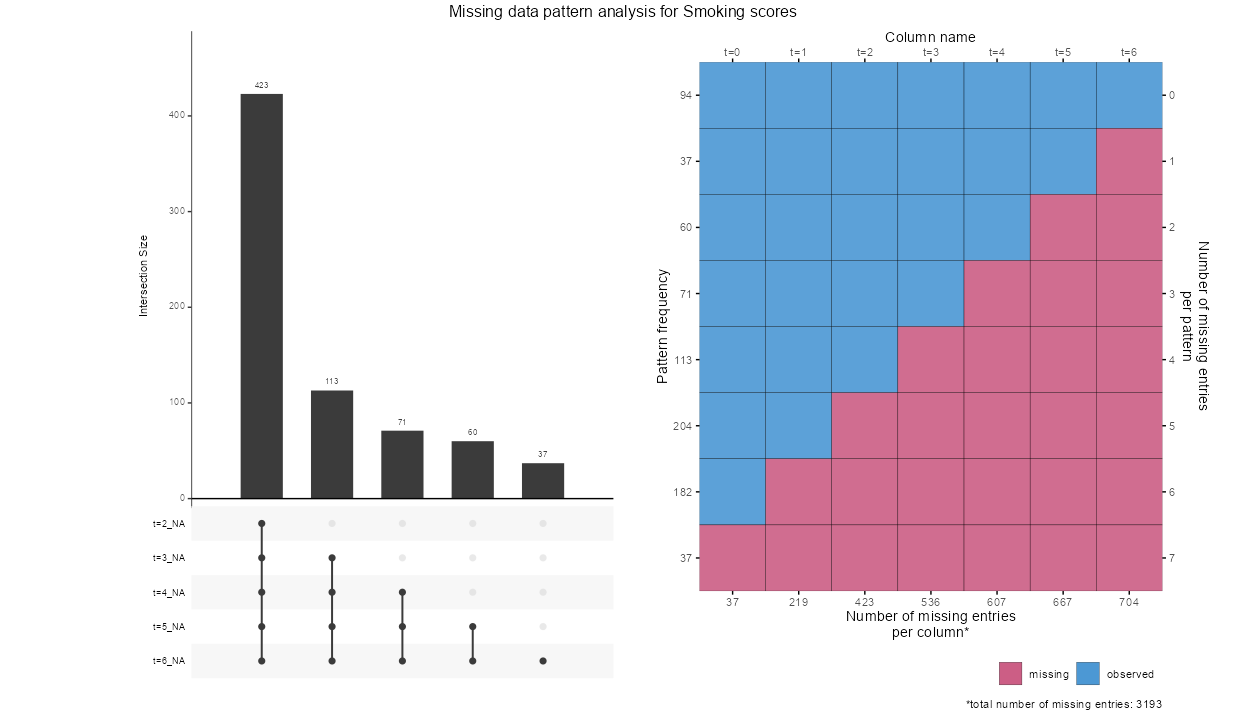

Supplement: Multimedia Appendix 1 [file formative_v9i1e64239_app1.zip › S35 Missing pattern analysis Smoking.png]

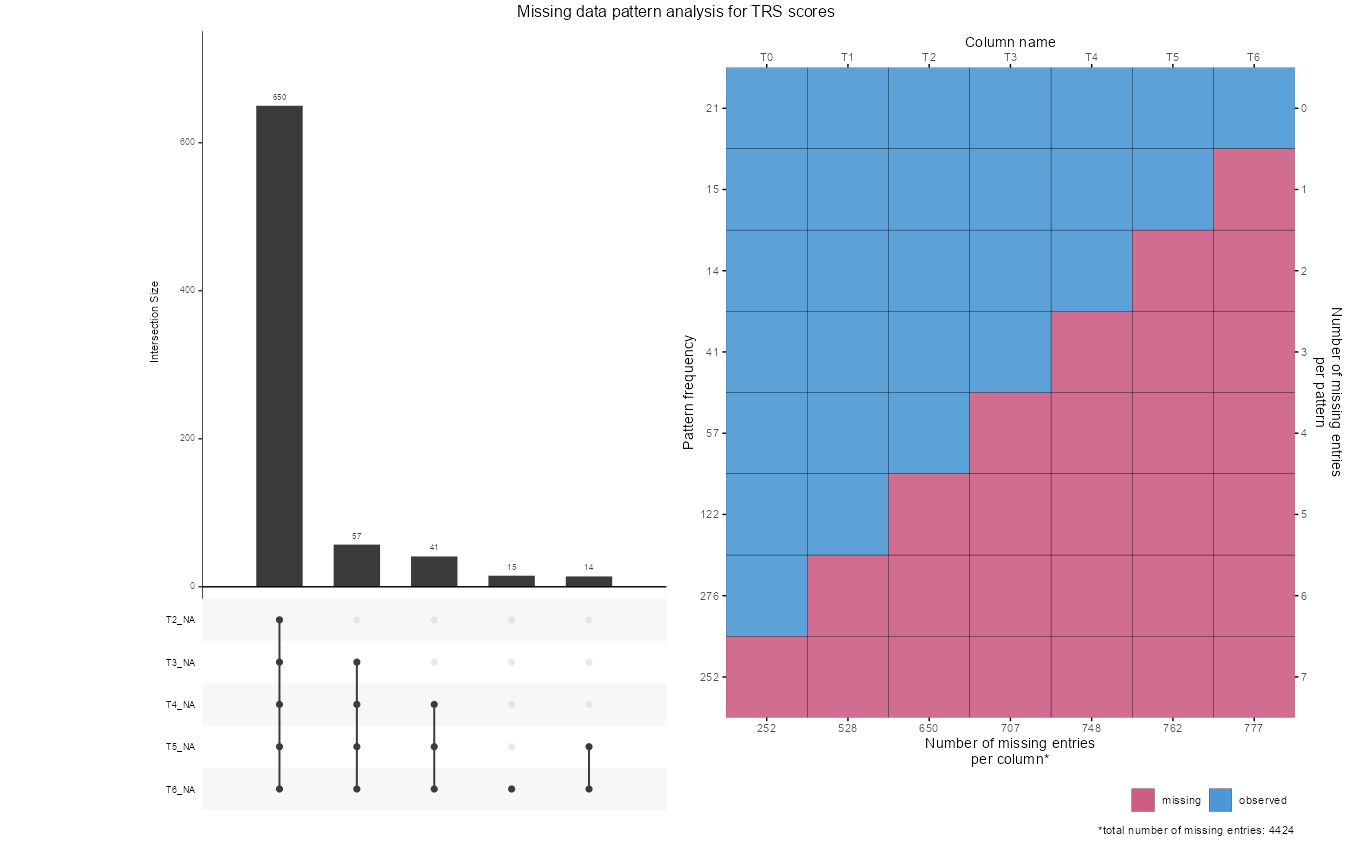

Supplement: Multimedia Appendix 1 [file formative_v9i1e64239_app1.zip › S36 Missing pattern analysis TRS.png]
