# Supplementary material for: Evaluation of the MyFertiCoach Lifestyle App for Subfertile Couples: Single-Center Evaluation of Augmented Standard Care
Source: JMIR Form Res. 2025 Mar 10;9:e64239. doi: 10.2196/64239 (PMC11933746; doi:10.2196/64239)
Supplement: Multimedia Appendix 2 [file formative_v9i1e64239_app2.docx]

Effectiveness of the MyFertiCoach lifestyle app for subfertile couples: a single center study

Statistical Analysis Plan

Contents

[Aim 2](#_Toc159689661)

[Context 2](#_Toc159689662)

[Data collection 2](#_Toc159689663)

[Primary and secondary outcome(s) 4](#_Toc159689664)

[Statistical analysis 5](#_Toc159689665)

[Descriptives 7](#_Toc159689666)

[Aggregations and transformations 8](#_Toc159689667)

[Missing data 8](#_Toc159689668)

[Missing data pattern analysis 8](#_Toc159689669)

[Multiple imputation 9](#_Toc159689670)

[Data analysis 10](#_Toc159689671)

[Primary outcome 10](#_Toc159689672)

[Secondary outcomes 11](#_Toc159689673)

[Sensitivity analyses 13](#_Toc159689674)

[Multiple imputation in case of MNAR 13](#_Toc159689675)

[Analysing non-numerical outcomes 14](#_Toc159689676)

[Statistical package used 15](#_Toc159689677)

# Aim

The aim of this Statistical Analysis Plan (SAP) is to describe in clear plain language how the data will be analyzed to answer the hypotheses posed prior to analysis. In short, a SAP describes any transformation and analysis done on the data, how to deal with missing data, and which mathematical models are used to analyze the primary and secondary outcome(s).

## Context

The MyFertiCoach (MFC) lifestyle app is developed to assess the nutritional behavior and lifestyle of couples. By using parameters such as BMI, smoking, nutrition, alcohol and stress, a personal coaching program is prepared which consists of several lifestyle modules (e.g., dietary advice, exercise, smoking, weight loss and/or mindfulness). During the registration process, baseline demographic parameters and questionnaires about dietary and lifestyle habits are completed.

# Data collection

The program length is set at six months, and progress is tracked via a monthly request for filling out several patients reported experience measures (PREMs) and patient reported outcome measures (PROMs). Patients are free to complete the questionnaire at their earliest convenience but need to provide a baseline measure to be included in the study. The specific information collected is based on a schematic which is shown in ***Figure 1*** and further described in ***Table 2***. The app is intended to be tailor made, which means patients may receive different questions based on their preferences and/or baseline answers. From baseline until six months, patients are asked to fill out a set of questionnaires for a total of seven times.


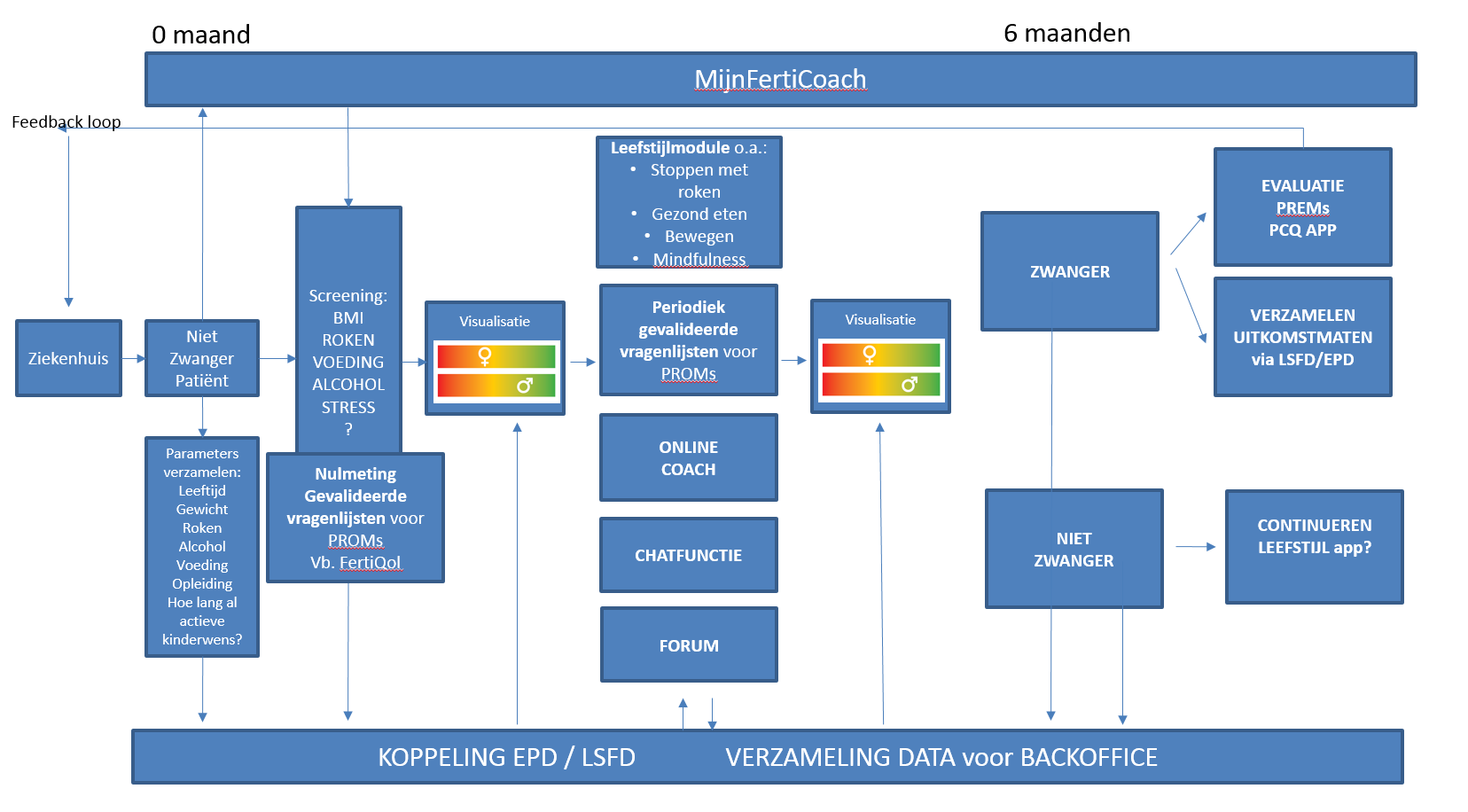


**Figure 1.** Schematic showing the type of data collected, and when. The specific questions being asked depends on the time of request and if a specific module has been included.

**Table 1.** Type of parameters collected, and when.

| **Parameter** | **Type** | **Baseline** | **Monthly^[[1]](#footnote-1)^** | **6 months**  **follow-up** | **2+ years follow-up** |
| --- | --- | --- | --- | --- | --- |
| Age | Numerical | X |  |  |  |
| Heritage | Nominal | X |  |  |  |
| BMI (weight/length^2^) | Numerical / Ordinal | X | X | X |  |
| Education | Ordinal | X |  |  |  |
| Smoking | Numerical /  Binary / Ordinal | X | X | X |  |
| Alcohol use | Numerical /  Binary / Ordinal | X | X | X |  |
| Drugs / anabolic steroids | Binary / Text | X | X | X |  |
| Folic acid | Binary | X |  |  |  |
| Vitamin D | Binary | X |  |  |  |
| Other vitamins / supplements | Binary / Text | X |  |  |  |
| Nutritional composition | Numerical | X | X | X |  |
| Activity | Numerical /  Binary / Ordinal | X | X | X |  |
| Screening for Distress and Referral Need | Numerical /  Nominal | X | X | X |  |
| Patient Activation Measure | Numerical /  Binary / Ordinal | X |  | X |  |
| FertiQol | Numerical | X |  | X |  |
| PCQ – Infertility | Numerical /  Binary / Ordinal |  |  | X |  |
| Treatments received | Nominal |  |  |  | X |
| Trajectory time | Datetime |  |  |  | X |
| Life birth | Binary |  |  |  | X |
| Healthy pregnancy | Binary |  |  |  | X |
| Healthy child | Binary |  |  |  | X |

## Primary and secondary outcome(s)

The data are transformed into various (composite) parameters of which the Rotterdam Reproduction Risk (R3) score, and the Preconception Dietary Risk (PDR) score, are the most important.

The **primary** outcome is the change in Total Risk Score (TRS) at three- and six-months follow-up. The TRS per individual is defined as the sum of all risk scores per behaviour (i.e., based on vegetable/fruit/folic acid intake, smoking, and alcohol use after three and six months). The higher the TRS, the unhealthier the nutrition and lifestyle habits that are present. An important distinction to make is that the TRS are calculated separately for females and their partners (male/female). Although both scores are numerical, their calculation and range differ (***Figure 2***).

The **secondary** endpoints are changes in BMI, activity score, PDR score, LAST-score, smoking habits, alcohol intake, and program adherence. The calculation of the PDR score is based on six questions which can only be answered with either yes or no (***Table 2***). Hence, the range of the PDR is 0-6 and a higher score is a better score. Although the PDR looks like a numerical variable, it is ordinal (scales with a range smaller than 10 often create mathematical problems when analysing them in a numerical way). Any ordinal scale can be transformed into a binary scale, but the categories must make sense (see **Statistical analysis**).

**Figure 2.** The scoring cards for females (left) and partners (right) when calculating the R3 score.


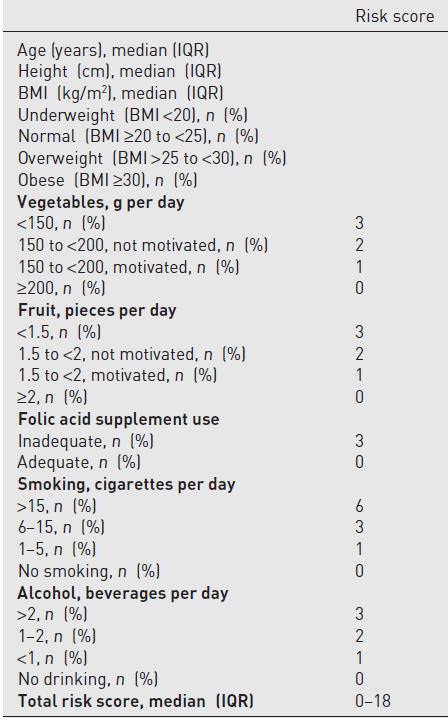

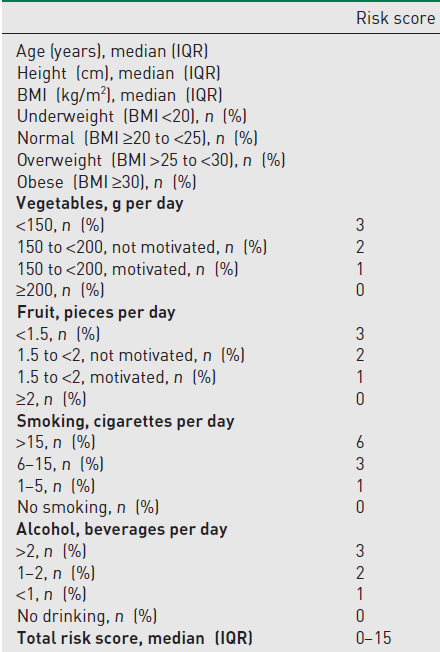


**Table 2.** Preconception Dietary Risk assessment.

| **PDR-item** | **Score** |
| --- | --- |
|  |  |
| At least four slices of whole wheat bread daily (or comparable servings of cereals) | 0-1 |
| The use of monounsaturated or polyunsaturated oils | 0-1 |
| ≥200 g of vegetables daily | 0-1 |
| ≥2 pieces of fruit daily | 0-1 |
| ≥3 servings of meat or meat replacers weekly | 0-1 |
| ≥1 servings of fish weekly | 0-1 |

# Statistical analysis

**Table 1** shows that we have multiple sources of information which provide information on different scales: *numerical*, *binary*, *nominal*, *ordinal* or *text*. Each of these types of information have different characteristics which need to be considered when describing and analysing the data (***Table 3***).

**Table 3.** Description of various data types.

| **Type** | **Description** |
| --- | --- |
|  |  |
| Nominal | Parameter with multiple options which have no natural order (e.g., warm/cold; sea/land). |
| Binary | Parameter with two options which have no natural order (e.g., yes/no; true/false). |
| Ordinal | Parameter with multiple options which have a natural order (e.g., best/better/good/worse/worst). |
| Numerical | Parameter which has a natural order and on which standard arithmetic such as subtraction or multiplication can be applied. |
| Text | Text |

For many of the parameters mentioned, information can be transformed. For instance, the number of steps walked during the day is a numerical parameter which can be transformed into a binary (‘active’ / ‘non-active’) or ordinal (0;1;2;3;4) scale. On the other hand, a binary variable cannot be made into a numerical variable because there are only two categories. This does not mean that we cannot implement models on these types of data, but it does not mean that the type of model used is highly dependent on the type of data collected (or the transformation applied). This is also the reason why for some ordinal variables, such as those coming from PROMs, transformations have been devised to create a numerical variable from an ordinal variable. A parameter which consists of text is not easily transformed into information suitable for arithmetic (although possible) but does nevertheless provide important contextual information in its raw form. Hence, we will add any textual information in the descriptive part of the analysis.

In the parts below, we will describe how we will deal with the specific **types** of information collected (except text). Mathematically it does not matter if a numerical score comes from questionnaire A or B because both questionnaires adhere to the same mathematical properties (which come from the numerical nature of the parameter) and thus need to be checked in the same manner.

Hence, in this statistical analysis plan, we will describe our plan for describing and transforming the data, how to deal with missing data, and which models we will use to answer the primary and secondary endpoint(s).

Finally, even the best and most thought-out SAP cannot plan for unforeseen insights. Hence, this SAP is set in place to describe the must-do analyses to answer the research question. Any other statistical analyses which we will conduct in the end, but which are not pre-specified here, can be deemed exploratory at best.

## Descriptives

First and foremost, we will provide descriptive statistics (descriptives) for each of the parameters of interest. For some parameters, such as length and weight, this means that we will provide both the descriptives for each of the parameters, and the derived BMI parameter. This is because both are numerical.

Contrary to popular belief it is not necessary to assess if variables are normally distributed. This is because most models require the residuals of the model to be normally distributed, not the raw data itself. However, it could very well be that the data are so skewed that this will impact the statistical model of choice. Although several descriptives, such as kurtosis^[[2]](#footnote-2)^, are available to describe the nature of the distribution, they are not easy to interpret. Hence, we will use the mean, median, variance, and range to describe numerical variables (**Table 4**). The higher the deviation between the mean and the median, the more skewed the data is. Combined with the variance and the range, we can describe the fatness of the distribution and the position of the tails. In the case of extremely skewed data, we will select a different statistical model which is described later in this SAP. Variables that are nominal, binary, or ordinal will be described in terms of their range, and frequency. Information coming from text can only be described in terms of frequency. For each parameter, we will also show the number of missing (both number and percentage).

**Table 4.** Descriptive statistics for each type of parameter.

| Type | Information shown | | | | | |
| --- | --- | --- | --- | --- | --- | --- |
|  | | | | | | |
|  | Mean | Median | Variance  *standard deviation* | Range  *min-max* | Frequency  *percentage* | Number of missing  *number; percentage* |
| Nominal |  |  |  | X | X | X |
| Binary |  |  |  | X | X | X |
| Ordinal |  |  |  | X | X | X |
| Numerical | X | X | X | X |  | X |
| Text |  |  |  |  | X | X |

### Aggregations and transformations

The parameters collected will be aggregated into known composites (such as BMI) or transformed if the specific manual of a questionnaire requires us to do so. Every parameter that is a derived parameter will also follow the manual included on how to transform and aggregate the data, but also on how to deal with missing data when transforming/aggregating.

## Missing data

A very important part of collecting descriptives is also to collect the number of missing data (**Table 4**). There is no official cut-off on what constitutes an acceptable or non-acceptable level of missingness. In addition, it is seldom clear why the data is missing, except for when someone has died. Hence, we not only need to explore the extent of missingness, but also its specific pattern. In the end, it is the pattern that warrants a change in analysis.

### Missing data pattern analysis

A pattern analysis means that we will collect for each parameter separately the amount of missing and ascertain how often multiple parameters are missing together. We will look for missing patterns across all items in the dataset. This can easily lead to an explosion of unique patterns (e.g., 100 items lead to 9.332622e+157 unique patterns). However, it is seldom the case that we will extract many missing patterns. In most cases, pattern analysis can be summarized in five-to-ten unique patterns. Keep in mind that the patterns derived do not provide unequivocal proof for a specific type of missing. However, we will try to classify them in three accepted types of missing data patterns: *missing completely at random*, *missing at random* or *missing not at random* (***Table 3***).

**Table 5.** The three types of missing data explained.

| **Pattern of missingness** | **Explanation** |
| --- | --- |
|  |  |
| Missing completely at random (MCAR) | The reason for missingness has no relationship with any of the other parameters included, nor the outcome(s) of interest. |
| Missing at random (MAR) | The reason for missingness can be explained by use of the other parameters included, or the outcome(s) of interest. |
| Missing not at random (MNAR) | The reason for missingness is dependent on the score for a particular parameter or outcome. Its explanation for missing lies within the score that is missing. |

Of the three possible patterns, the MNAR is truly problematic. For instance, if a couple does no provide information on their lifestyle because their lifestyle is too bad to fill out the questionnaire, the missingness of the score is of importance and thus something that cannot be ignored but also cannot be modelled. This is because the missingness in MNAR is missing for a reason, which has to do with the actual score someone could have provided but did not. If we would analyse the dataset as is (complete case analysis) we would end up having estimates of effect that are not internally valid, nor externally applicable. In contract to the MAR pattern, we can also not use other parameters included, since there is no proxy.

We must only change models when a missing data pattern is MNAR. For the rest, we can deal with missingness using multiple imputation.

### Multiple imputation

Missing data can theoretically be left unchecked if the data is missing completely at random (MCAR) as this will not lead to systematic bias. However, a loss in data also means a spread in variance and hence a loss in power to look for statistical significance. Therefore, we aim to complete the dataset using multiple imputation which means that we will fill the holes with non-observed but modelled data. The use of missing imputation to fill data gaps is not new and an accepted way of completing a dataset, although not without caveats^[[3]](#footnote-3)^. Because the data is not observed we can never be sure if the imputation is correct. This is a very important distinction to make because the “gut feeling” approach would be to compare the imputed values with the observed values and deem them “correct” if they have a large overlap. However, we can never be certain what the value would be. Hence, the proper way to impute the data whilst simultaneously safeguard against the introduction of new bias is by:

1. building a multiple imputation model,
2. checking the model for accuracy and validity^[[4]](#footnote-4)^,
3. using the model to create ten imputed datasets,
4. apply the statistical model of interest in each dataset,
5. combing all the results and obtain a weighted result,
6. compare the weighted result with the results coming from the dataset with missing observations.

In summary, this process entails that the gaps in the original dataset need to be filled by a model built to discern why data is missing, and then use that information to estimate the highest likelihood of observing a specific non-observed value. This is done multiple times, combined, and then compared with the original dataset. In the end, this process enables us to use all observed information, and the most likely substitute for the non-observed values.

Once again, this only works if the missing data pattern is MCAR or MAR. For MNAR, such analysis is not feasible because we will never have the necessary information to model what the likely value would have been – that information is dependent on the information we are trying to model which is missing. We will address this possibility later.

##

## Data analysis

Once the data has been transformed, aggregated and the missing data pattern analysis has been made, it is time to analyse the data according to the primary and secondary outcomes. In this part we will also describe how to include the multiple imputation which is partly bound to the statistical procedure chosen to analyse the data.

### Primary outcome

The main objective of the study is to investigate the effect of following the MFC app program on the change in Total Risk Score (TRS) after three and six months. Using a maximum of seven measurements per patient (one baseline measurement and six follow-up measurement), and in the absence of a clinically relevant cut-off-value, we will analyse the change in TRS using a (*hierarchical) linear mixed-model (LMM)^[[5]](#footnote-5)^*. Linear mixed models are regression models that are designed to analyse longitudinal data and can deal with MCAR and MAR data^[[6]](#footnote-6)^.

When analysing longitudinal data, you must consider that observations are not unique because they are coming from the same person. Hence, they are correlated, which decreases the effective sample size of the data^[[7]](#footnote-7)^. This influences the variance estimates which in turn influences the power for statistical significance. Also, the baseline value is an important anchor from which to estimate change.

Hence, to estimate the change in TRS we will build several LMMs with either a *random intercept*, *random slope* or *random intercept and random slope*. A model with only a random intercept assumes that patients differ substantially in their baseline value, but not in their improvement. A random slope model assumes that each patient starts at almost the exact same baseline value, but the trajectories differ (i.e., some improve, some do not, and at different times). A random intercept random slope models assumes that patients not only differ in their starting value but also in their trajectories. This last model, if chosen, would show us that the effect of the app is different in a study group that is already different to begin with.

Assessment of each model for accuracy and parsimony will be done using the F-test (ANOVA)^[[8]](#footnote-8)^ and the Aikaki Information Criterion (AIC)^[[9]](#footnote-9)^. Model assumptions will be tested by use of graphs and visualizations.

An important note to make is that we will not conduct separate analyses for three and six months. We will build a single model from which the statistical significance of change can be derived at any moment in time until the last time observed. The primary focus will be three and six months, and we will derive estimates and statistical significance by comparing the observed and predicted values, and by plotting the effect size of change for each month during the six-month follow-up. In the end, the final LMM of choice will be able to estimate the change in TRS at three and six months, even if the questionnaires have not been filled at three of six months specifically. To properly include time we will use linear, polynomial, or natural cubic splines^[[10]](#footnote-10)^. Statistical significance is an estimate which does not contain a zero in its 95% confidence interval^[[11]](#footnote-11)^.

### Secondary outcomes

We analyse the secondary outcomes (i.e., BMI, Activity score, PDR score, LAST-score, Smoking habits, Alcohol intake) like the primary outcome, except for *program adherence*. The only predictor included would be time to determine the effect of change and when it occurred.

#### Program adherence

The assessment of adherence is a TRUE/FALSE at each time-point or can be a datetime variable (time from start till to last entry). In either case, we can assess adherence by use of a time-to-event model (in which the event is non-adherence). This is also called survival analysis^[[12]](#footnote-12)^, and several models are available^[[13]](#footnote-13)^. For our analysis, we will use the straightforward Kaplan-Meier to determine the failure rate^[[14]](#footnote-14)^. This will also allow us to look for statistically significant differences in failure-rate curves between groups. A Cox Regression model (or proportional hazard model)^[[15]](#footnote-15)^ will be built to determine the influence of multiple demographic and clinical variables on failure (which is also called a hazard rate – or the risk of having an event at a specific time-point based on multiple predictors). A Kaplan-Meier does not have specific statistical assumptions, but a Cox-Regression model. We will test every assumption (i.e., proportionality of hazards, linearity of predictors) using graphs and visualizations.

### Sensitivity analyses

The above stated analysis of the primary and secondary outcome(s) will lead to seven different models, with no predictors included except time. This raises issues in terms of spurious results, as all have the same 5% false-positive included when determining statistical significance. To safeguard against spurious results, it is important to assess if the seven outcomes are correlated. We will do this by creating a correlation matrix, and then by building a multivariate model^[[16]](#footnote-16)^ which will assess the role of time for all outcomes simultaneously. This way, we can determine which outcomes are similarly affected by time and thus share common variance (because they are proxies). This can only be done for the outcomes that are numerical, not for the *program adherence outcome*. If correlations are found (larger than 0.05) between outcomes, we will include correlated outcomes as predictors in the original LMM models. For instance, the primary outcome model of TRS will now not only include time, but also the secondary outcome BMI as a predictor. We will then also start to include other demographical and clinical variables extracted at baseline. To assess the appropriateness of these inclusions, we will consecutively drop parameters from a fully specified fixed-effects model using the Chi-squared test^[[17]](#footnote-17)^. This means including both main effects and interaction effects. The final model will be assessed for appropriateness in the exact same way as stated in the *Primary outcome* section. This way, we can test the main effect of the app using multiple models.

### Multiple imputation in case of MNAR

In case of MNAR we will not be able to conduct multiple imputation. First, we will assess if the missing data pattern is linked to *program adherence*. If data is missing following a stop in program adherence, this could mean that the explanation for non-adherence can be found in the missing scores themselves. Although it could very well be that patients show intermittent missingness (which is most likely the case in a MAR situation and thus tackled by use of a MAR model or multiple imputation model), a sudden stop is a strong indication for a MNAR response. Which is problematic for our analysis if it happens before three- or six-months follow-up. Hence, we will create a joint model, which is a combination of a LMM and a survival model. Although rather new^[[18]](#footnote-18)^, it is accepted as a viable replacement of older models such as pattern-mixture modelling^[[19]](#footnote-19)^. We have such models in the past when analysing Quality-of-Life changes in a population of palliative pancreas patients^[[20]](#footnote-20)^. The end-result of a joint model is (1) a survival model on program adherence, which lends information from the LMM model, and (2) the LMM model which adjusts its estimates based on the survival model.

### Analysing non-numerical outcomes

All the above, except for program adherence, has focused on numerical outcomes (parameters). However, **Table 1** shows that most numerical parameters can be transformed into ordinal or binary parameters. In general, it is not advised to transform as this will lead to a loss in information and power. Hence, we will only downscale a numerical variable into an ordinal or binary variable if the original numerical variable is extremely skewed. For instance, if a scale (which goes from 0-10) has an abundant number of zero’s it is virtually impossible to meaningfully transform the data since zero scores cannot be log-transformed nor exponentiated, which are standard methods for dealing with highly skewed data.

To analyse these datasets, we will use *Generalized Linear Mixed Models* (GLMMs)^[[21]](#footnote-21)^ – a family of models which can accommodate both binary and ordinal models through link functions^[[22]](#footnote-22)^. In short, the transformation of the variables happens within the model, instead of prior to modelling. This enables the use of far larger range of models, some of which can deal with inflated zeros for instance. The assessments for a GLMM differs from that of a LMM because of a difference in statistical assumptions (for instance, the residuals are not expected to be normal). Based on the specific type of model used, we will assess assumptions by use of graphs and visualizations. Comparisons between models will be conducted like the LMM models. The end result is a list of models, comprised of important predictors, to assess the primary and secondary outcome(s) of interest.

## Statistical package used

All analyses will be done using the R statistical package^[[23]](#footnote-23)^ which is a widely known and accepted software program. The specific libraries used cannot be pre-specified but will be listed in the publication and made visible in the coding files.

1. If a specific lifestyle module has been included [↑](#footnote-ref-1)
2. https://en.wikipedia.org/wiki/Kurtosis [↑](#footnote-ref-2)
3. https://www.bmj.com/content/338/bmj.b2393 [↑](#footnote-ref-3)
4. using likelihood and root mean squared error (RMSE) [↑](#footnote-ref-4)
5. https://en.wikipedia.org/wiki/Mixed_model [↑](#footnote-ref-5)
6. https://medium.com/mlearning-ai/introduction-to-mixed-models-in-r-9c017fd83a63 [↑](#footnote-ref-6)
7. e.g., a dataset containing 10 perfectly correlated observations has an effective sample size of 1 [↑](#footnote-ref-7)
8. https://en.wikipedia.org/wiki/F-test [↑](#footnote-ref-8)
9. https://en.wikipedia.org/wiki/Akaike_information_criterion [↑](#footnote-ref-9)
10. https://en.wikipedia.org/wiki/Spline_(mathematics) [↑](#footnote-ref-10)
11. https://en.wikipedia.org/wiki/Confidence_interval [↑](#footnote-ref-11)
12. https://en.wikipedia.org/wiki/Survival_analysis [↑](#footnote-ref-12)
13. https://blog.devgenius.io/survival-analysis-in-sas-kaplan-meier-cox-regression-time-varying-predictors-recurrent-events-4ae7cd95f8c0 [↑](#footnote-ref-13)
14. https://en.wikipedia.org/wiki/Kaplan%E2%80%93Meier_estimator [↑](#footnote-ref-14)
15. https://en.wikipedia.org/wiki/Proportional_hazards_model [↑](#footnote-ref-15)
16. https://www.ncbi.nlm.nih.gov/pmc/articles/PMC3518362/ [↑](#footnote-ref-16)
17. https://en.wikipedia.org/wiki/Chi-squared_test [↑](#footnote-ref-17)
18. https://bmcmedresmethodol.biomedcentral.com/articles/10.1186/s12874-018-0592-9 [↑](#footnote-ref-18)
19. https://bmcmedresmethodol.biomedcentral.com/articles/10.1186/s12874-018-0639-y [↑](#footnote-ref-19)
20. https://pubmed.ncbi.nlm.nih.gov/37647972/ [↑](#footnote-ref-20)
21. https://en.wikipedia.org/wiki/Generalized_linear_mixed_model [↑](#footnote-ref-21)
22. https://pub.towardsai.net/generalized-linear-mixed-models-in-sas-distributions-link-functions-scales-overdisperion-and-4b1c767bb89a [↑](#footnote-ref-22)
23. https://www.r-project.org/ [↑](#footnote-ref-23)
